# Experimental and theoretical insights into the adsorption mechanism of methylene blue on the (002) WO_3_ surface

**DOI:** 10.1038/s41598-024-78491-3

**Published:** 2024-11-06

**Authors:** Khaoula Hkiri, Hamza Elsayed Ahmed Mohamed, Mohamed Mahrous Abodouh, Malik Maaza

**Affiliations:** 1 UNESCO UNISA Africa Chair in Nanoscience and Nanotechnology, College of Graduate studies, University of South Africa, Pretoria, South Africa; 2 Energy Materials Laboratory, Physics Department, School of Sciences and Engineering, The American University in Cairo (AUC), New Cairo, 11835 Egypt; 3 UNESCO UNISA Africa Chair in Nanosciences & nanotechnology, Pretoria, South Africa

**Keywords:** WO_3_ nanoflakes, Adsorption study, Molecular dynamic simulation, Methylene Blue, Materials science, Nanoscience and technology, Environmental chemistry, Catalysis, Chemical biology, Environmental chemistry, Green chemistry, Theoretical chemistry

## Abstract

This work investigates the efficiency of green-synthesized WO_3_ nanoflakes for the removal of methylene blue dye. The synthesis of WO_3_ nanoflakes using Hyphaene thebaica fruit extract results in a material with a specific surface area of 13 m^2^/g and an average pore size of 19.3 nm. A combined theoretical and experimental study exhibits a complete understanding of the MB adsorption mechanism onto WO_3_ nanoflakes. Adsorption studies revealed a maximum methylene blue adsorption capacity of 78.14 mg/g. The pseudo-second-order model was the best to describe the adsorption kinetics with a correlation coefficient (R^2^) of 0.99, suggesting chemisorption. The intra-particle diffusion study supported a two-stage process involving surface adsorption and intra-particle diffusion. Molecular dynamic simulations confirmes the electrostatic attraction mechanism between MB and the (002) WO_3_ surface, with the most favorable adsorption energy calculated as -0.68 eV. The electrokinetic study confirmed that the WO_3_ nanoflakes have a strongly negative zeta potential of -31.5 mV and a uniform particle size of around 510 nm. The analysis of adsorption isotherms exhibits a complex adsorption mechanism between WO_3_ and MB, involving both electrostatic attraction and physical adsorption. The WO_3_ nanoflakes maintained 90% of their adsorption efficiency after five cycles, according to the reusability tests.

## Introduction

Water pollution has emerged as a critical environmental issue, particularly with the increasing discharge of industrial effluents. Among these pollutants, wastewater from the textile industry is especially significant due to its high dye content. The widespread use of dyes, essential for various applications such as textiles, paints, and pigments, has led to the production of approximately 1.6 million tons annually, with a substantial portion being released untreated into water bodies^1^. This introduces harmful chemicals, including toxic heavy metals and carcinogens^2^, disrupts aquatic ecosystems, and poses serious health risks to humans, such as respiratory issues, skin irritation, and even cancer^1^. Effective removal of these pollutants from wastewater is crucial to prevent environmental degradation and protect public health^3^. Various methods have been employed to address dye contamination, including adsorption^3^, photocatalysis^4^, self-flocculating^5^, sonochemical degradation^6^ and electrochemical treatments^7^. Among these, adsorption remains the most favored approach due to its simplicity and effectiveness, with commercial activated carbon being a common choice due to its high surface area and porosity (500–2000 m^2^/g)^3^. Despite its effectiveness, the high cost of commercial activated carbon and the expense associated with its regeneration have driven the search for more cost-effective alternatives that can be used more economically on a large scale^8–10^.

Methylene blue (MB) is a synthetic dye used in various industries, such as textiles and printing, as well as in medical treatments for methemoglobinemia. However, MB is also an environmental concern due to its persistence and potential toxicity, which necessitates proper wastewater treatment^11^. In this context, adsorption processes have been reported as effective methods for removing methylene blue using various adsorbents^3,12,13^. Different types of biological materials, including macroalgae and bacterial species, have been investigated for their effectiveness in removing MB from aqueous solutions^14^.In addition, hydroxyapatite-based nanocomposites have demonstrated a high adsorption capacity for dye pollutants^15^. Several previous reports have also documented the efficiency of many other organic and inorganic materials in removing methylene blue by adsorption^9,15,16^. In addition to these materials, certain nanomaterials have demonstrated remarkable potential in adsorption applications. For instance, metal oxide nanoparticles (NPs), such as ZnO, WO_3_, SnO_2_, Fe_2_O_3_, TiO_2_, ZrO_2_, Co_3_O_4_, and others, have emerged as highly effective adsorbents due to their large surface area, quantum size effects, and unique structures, including variable pore sizes and low solubility^17,18^. They are cost-effective to synthesize, offer robust mechanical stability, and excel at adsorbing organic dyes through complex formation interactions. Metal oxides also feature rapid adsorption times, reusability, and serve a dual role as chemical adsorbents and biological disinfectants in wastewater treatment^17,18^. In particular, WO_3_ has shown noteworthy adsorption capacity for organic dyes. For instance, Morales et al. (2008) investigated the methylene blue adsorption on WO_3_ nanocrystalline and correlated the acidic characteristics of the WO_3_ surface with the adsorption process^19^. Building on this, Sangeeta et al. (2017) used the co-precipitation method to synthesize WO_3_ nanoparticles to test their adsorption efficacy toward methylene blue^20^. According to Sangeeta et al. (2017), the adsorption occurred because the negatively charged surface of WO_3_ attracted and interacted with the cationic dye^20^. In addition to methylene blue, colloidal WO_3_ particles have been shown to interact with a range of polymeric xanthene dyes such as RhB, Rh3B, Rh 19, Rh 6G, Rh 110 and Rh 123. This interaction occurs because the negatively charged WO_3_ particles attract the positively charged parts of the dye molecules^21^. However, most studies on the adsorption of organic dyes onto WO_3_ have used spherical tungsten oxide for methylene blue without deeply exploring the adsorption mechanism. These spherical particles were prepared using traditional chemical methods. Therefore, the aim of this study is to enhance methylene blue adsorption by using WO_3_ nanoflakes and to investigate the adsorption mechanism more thoroughly.

Over the years, researchers have successfully created various forms of nanostructured WO_3_, including nanoplates, nanosheets, nanoflakes, and nanowires, using both physical and chemical methods^22–26^. These methods involve processes such as hydrothermal reactions, anodization, sputtering, and chemical vapor deposition techniques. However, these approaches can be toxic and expensive due to the need for equipment and energy-intensive conditions. As a result, researchers are increasingly interested in eco-friendly methods for producing nanomaterials that take advantage of their compatibility. One approach involves utilizing biological waste materials, such as microorganisms, as various plant components like leaves, roots, and flowers. Recent research has shown that green synthesis methods can enhance dye adsorption by improving the properties of nanoparticles^27–30^. Similarly, extracts from Thebaica fruit, known for their antioxidant activity and metal ion binding properties, can significantly aid in regulating and stabilizing nanoparticles^31^. These extracts contain polyphenols that contribute to stabilizing and chelating properties, influencing the shapes, sizes, and structures of the nanomaterials^31^. In previous work, Thebaica fruit extracts were used to synthesize various materials, including ZnWO_4_ and CaZrO_3_^32–43^.

This work aim to enhance the adsorption capacity of methylene blue by using WO_3_ nanoflakes and green chemistry methods for nanoparticle synthesis. The synthesis methodology was explored, the adsorbent was characterized, and adsorption studies were conducted. In addition, the adsorption mechanism is thoroughly investigated by exploring the detailed interactions between methylene blue and WO_3_ using new methods. The theoretical insights offer valuable information about the adsorption sites, confirming the experimental findings.

## Materials and synthesis

### Chemicals

Sodium tungstate dehydrate (Na_2_WO_4_·2H_2_O, ≥ 99% purity, Sigma-Aldrich) and methylene blue (C_16_H_18_C_l_N_3_S·xH_2_O, >= 95% purity, Sigma-Aldrich) have been used without additional purification.

### Methodology for WO_3_ nanoflakes synthesis using hyphaene thebaica fruit extract

**Fig. 1:**
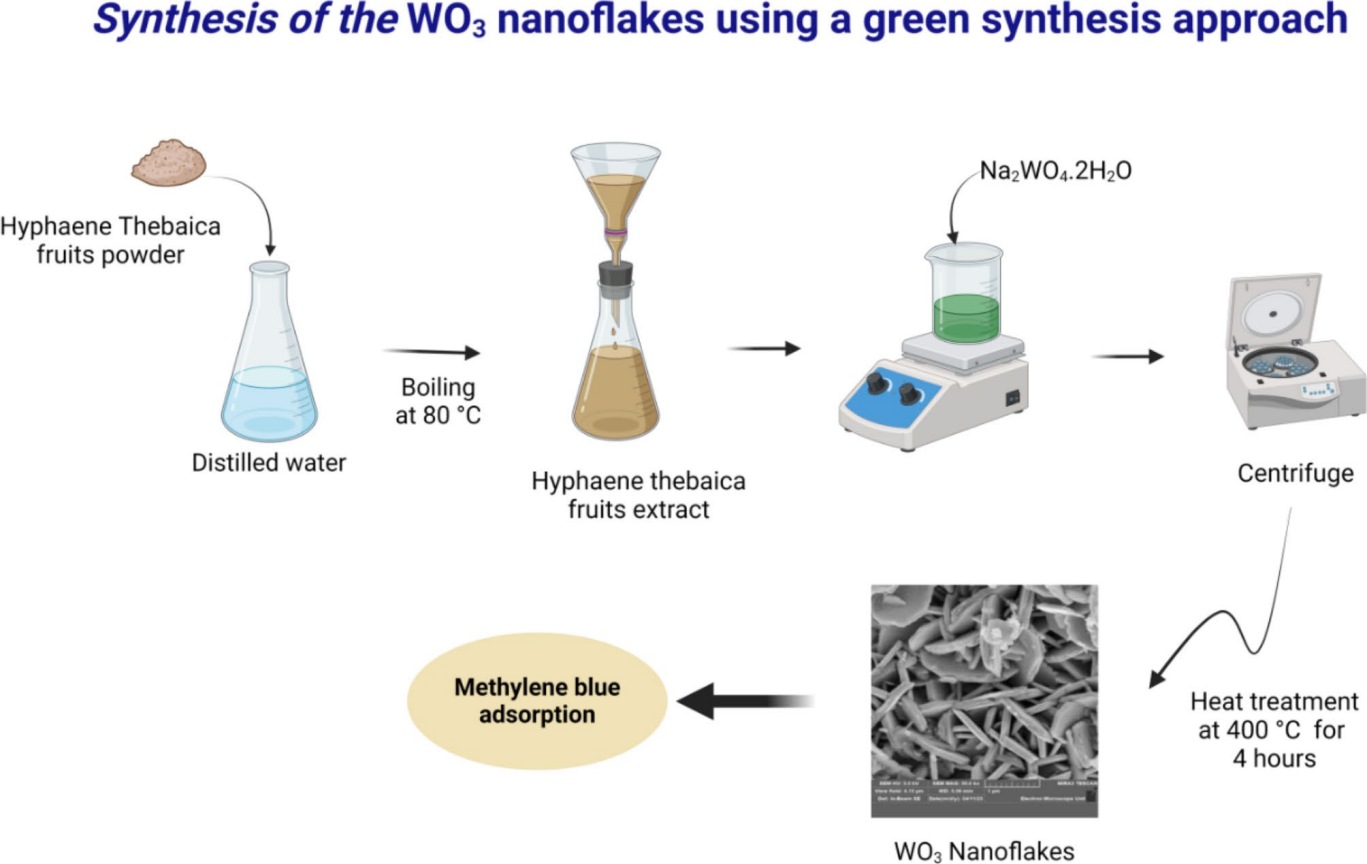
Schematic of the green synthesis route of WO_3_ nanoflakes.

Figure 1 shows a schematic illustration of the preparation of Hyphaene thebaica fruit extract and the biosynthesis process of WO_3_. The fruits of Hyphaene thebaica were collected from Egypt and processed through a rigorous preparation method. This involved washing the fruits with distilled water, drying them, and finely grinding them to obtain a powdered form. Next, 5 g of the powdered fruits were added to DI water, and the mixture was heated to 80 °C for two hours. After cooling the fruit extract to room temperature, Whatman paper was used to filter out any remaining plant material.

The synthesis of WO_3_ nanoflakes followed a green chemistry route by dissolving 6.10^− 3^ mol of tungsten precursor in 100 ml of the fruit extract. The mixture was stirred for 24 h at room temperature and then centrifuged to collect the precipitate. The obtained powder was washed, dried, and annealed at 400 °C for 4 h.

### Adsorption evaluation

In the adsorption kinetic experiment, different doses (0.1, 0.15, and 0.2 g) of tungsten trioxide were added to 200 ml of methylene blue (20 ppm as the initial dye concentration) at neutral pH. At selected times, samples were withdrawn and the suspensions were centrifuged. The residual dye concentration in the supernatant was determined by measuring the absorbance at the previously determined λ_max_ using a calibration curve. The results were averaged based on duplicate measurements. After each adsorption cycle, the WO_3_ nanoflakes were washed with ethanol and distilled water, followed by sonication to ensure complete removal of methylene blue. The nanoflakes were then heated at 200 °C for 2 h to regenerate the adsorbent. This process was repeated for five cycles with minimal loss in adsorption efficiency.

### Characterization techniques

WO_3_ crystallinity was determined using an X-ray diffractometer with CuKα as the source, operating under conditions of 40 mA and 40 kV. Raman measurements were performed using the Witec Confocal Raman Microscope (alpha300) with a 532 nm laser excitation. Energy-dispersive X-ray spectroscopy (EDX) was used to confirm the purity and composition of elements in WO_3_. Transmission electron microscopy (Technai Osiris TEM) and scanning electron microscopy (Tescan MIRA3 RISE SEM) are used to explore the morphology and particle shape and size of WO_3_. The optical properties of WO_3_ were carried out using a spectrophotometer (VARIAN, Cary 5000, USA) in the reflectance mode. A Micromeritics Tristar II 3020 surface area and porosity analyzer was used to analyze the nitrogen’s adsorption and desorption on the sample’s surface in order to calculate the specific surface area. The zeta potential and the particle size distribution of the sample were characterized using Malvern Zetasizer Nano ZS. Fourier transform infrared spectrophotometry was used to identify the functional groups in the materials prior to and following MB adsorption using the PerkinElmer Spectrum 400 FTIR/FT-NIR spectrophotometer.

## Characterization of WO_3_ adsorbent

### Structure and microstructure analysis

**Fig. 2:**
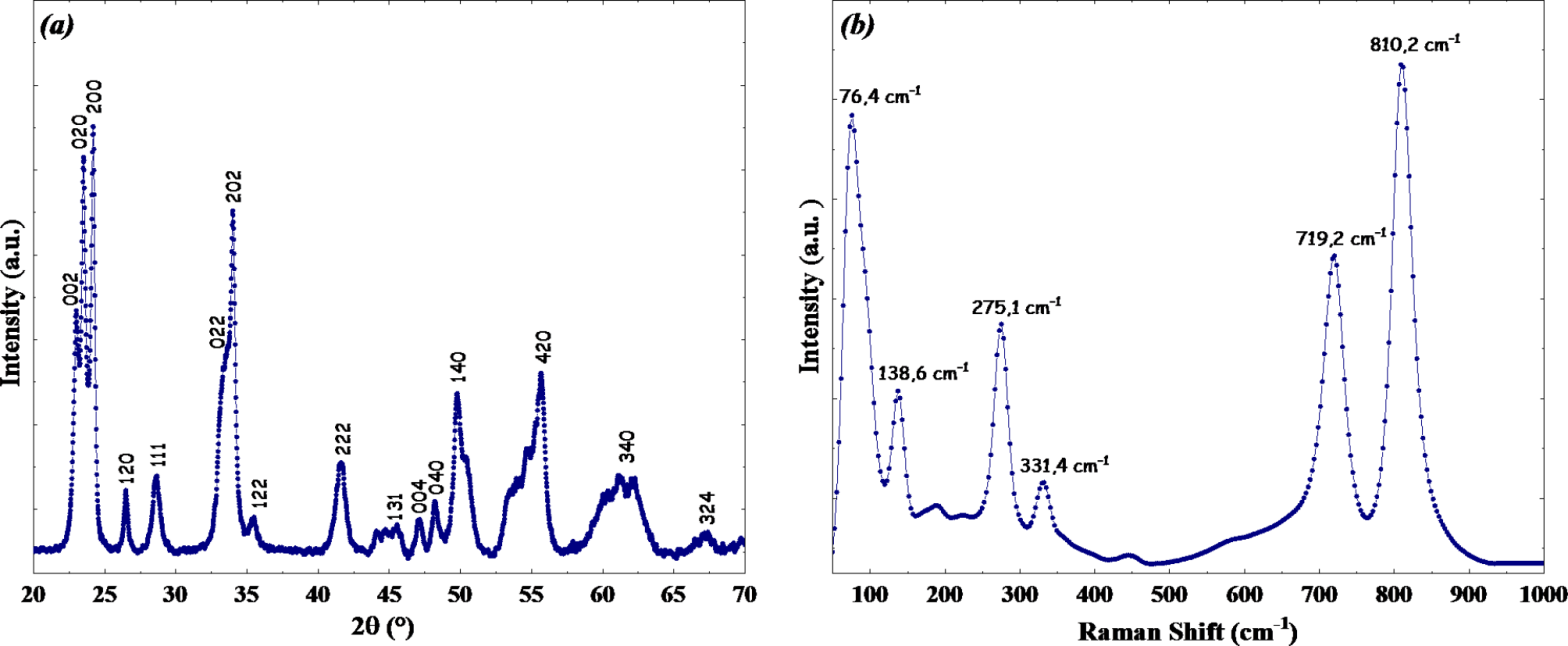
(**a**) X-Ray diffraction pattern, and (**b**) Raman spectrum of WO_3_ adsorbent.

Figure 2 displays the X-ray diffraction (XRD) pattern of the biosynthesized WO_3_ adsorbent. XRD analysis reveals peaks consistent with the WO_3_ monoclinic phase pattern (P2₁/c). Additionally, the pattern reveals no signs of impurities. The narrow and sharp peaks prove its high degree of crystallinity of the obtained powder.

**Fig. 3:**
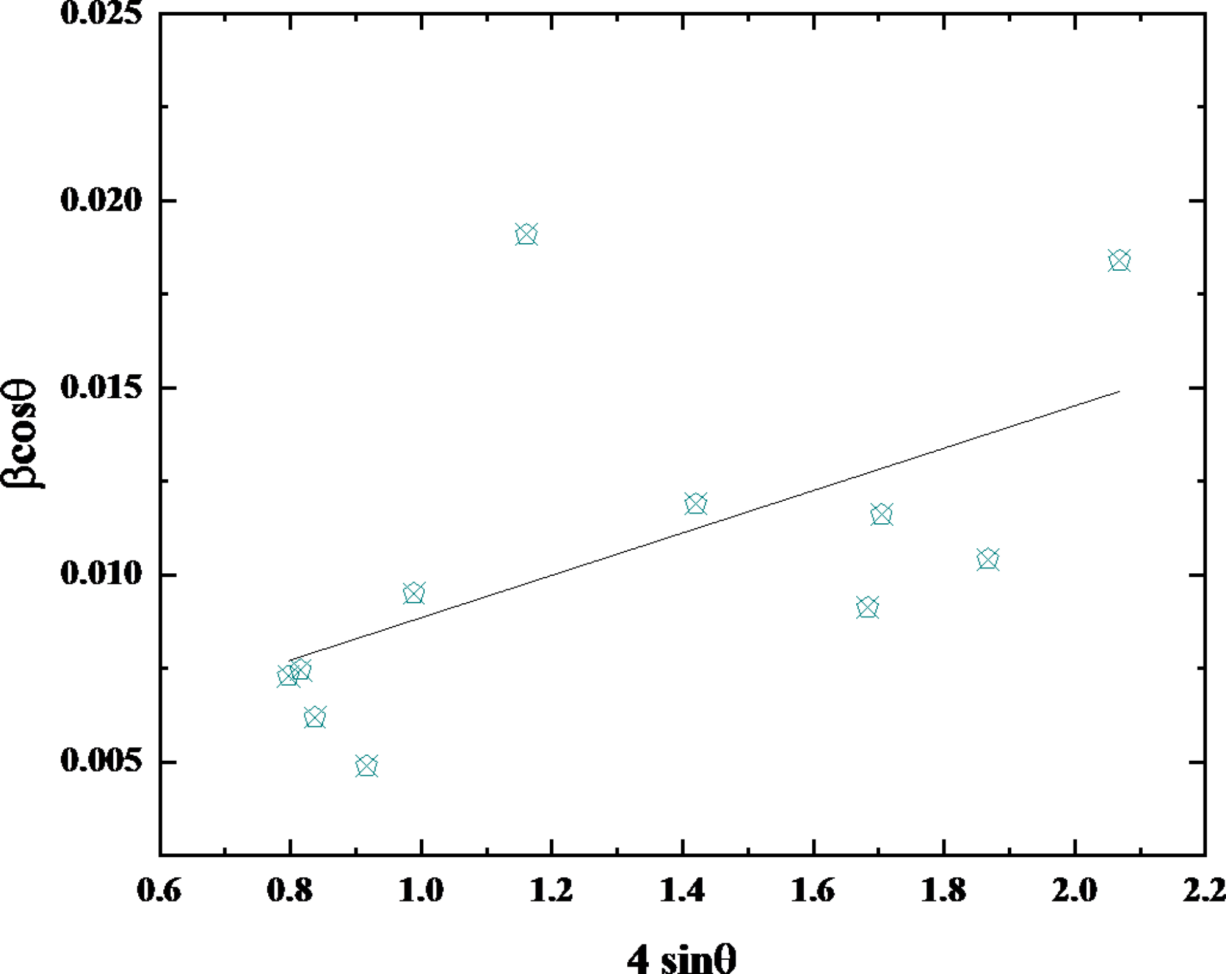
UDM plot for WO_3_ nanoflakes.

The average particle size and microstrain were estimated using the Williamson-Hall (W-H) method based on the uniform deformation model (UDM),

1 $$\beta \cos ~\left( \theta \right) = ~\frac{{k\lambda }}{D} + 4\varepsilon \sin \left( \theta \right)$$

Where$\:\:\beta\:$ is the full width at half of the maximum intensity for different diffraction planes. $\:D$ is the crystalline size in nanometers, $\:{\uplambda\:}$ is the wavelength of the radiation (1.5405 Å for CuKα radiation), $\:\text{k}$ is a constant equal to 0.94, and$\:\: \theta$ is the peak position.

Figure 3 plots the Williamson-Hall (W-H) equation, with $\:4\text{sin}\left( \theta\right)$ on the x-axis and $\:\beta\:cos\:\left( \theta\right)$ on the y-axis. The resulting straight line fits the data well, with a correlation coefficient R^2^ of 0.8861. The slope of this line reveals the intrinsic strain, while the intercept provides the average particle size of the WO_3_ nanoflakes. Size confinement mainly causes crystal lattice distortion in a nanoparticle, leading to expansion or contraction. Additionally, this confinement creates various lattice defects, contributing to lattice strains. The positive slope on the UDM plot indicates lattice expansion, which leads to intrinsic strain in the nanocrystals. The intrinsic strain was calculated as 0.53 × 10^− 2^ from the slope, and the crystallite size was determined to be 46.71 nm from the intercept of the straight line.

Raman spectroscopy was used to investigate the molecular vibrations in the produced WO_3_, and the results are presented in (Fig. 2b). The Raman spectrum shows distinct peaks at 137 cm^− 1^, 190.4 cm^− 1^, 273.3 cm^− 1^, 332.4 cm^− 1^, 716.4 cm^− 1^, and 813.7 cm^− 1^. For the stretching mode W-O-W, the peaks at 813.7 cm^− 1^ and 716.4 cm^− 1^ are specifically related to shorter and longer W-O-W bonds, respectively. The peaks at 137 cm^− 1^ and 190.4 cm^− 1^ are indicative of the WO_3_ crystallinity. Whereas those at 273.3 cm^− 1^ and 332.4 cm^− 1^ correspond to the W-O-W bending vibrations of bridging oxygen^44^.

### Surface morphology analysis

**Fig. 4:**
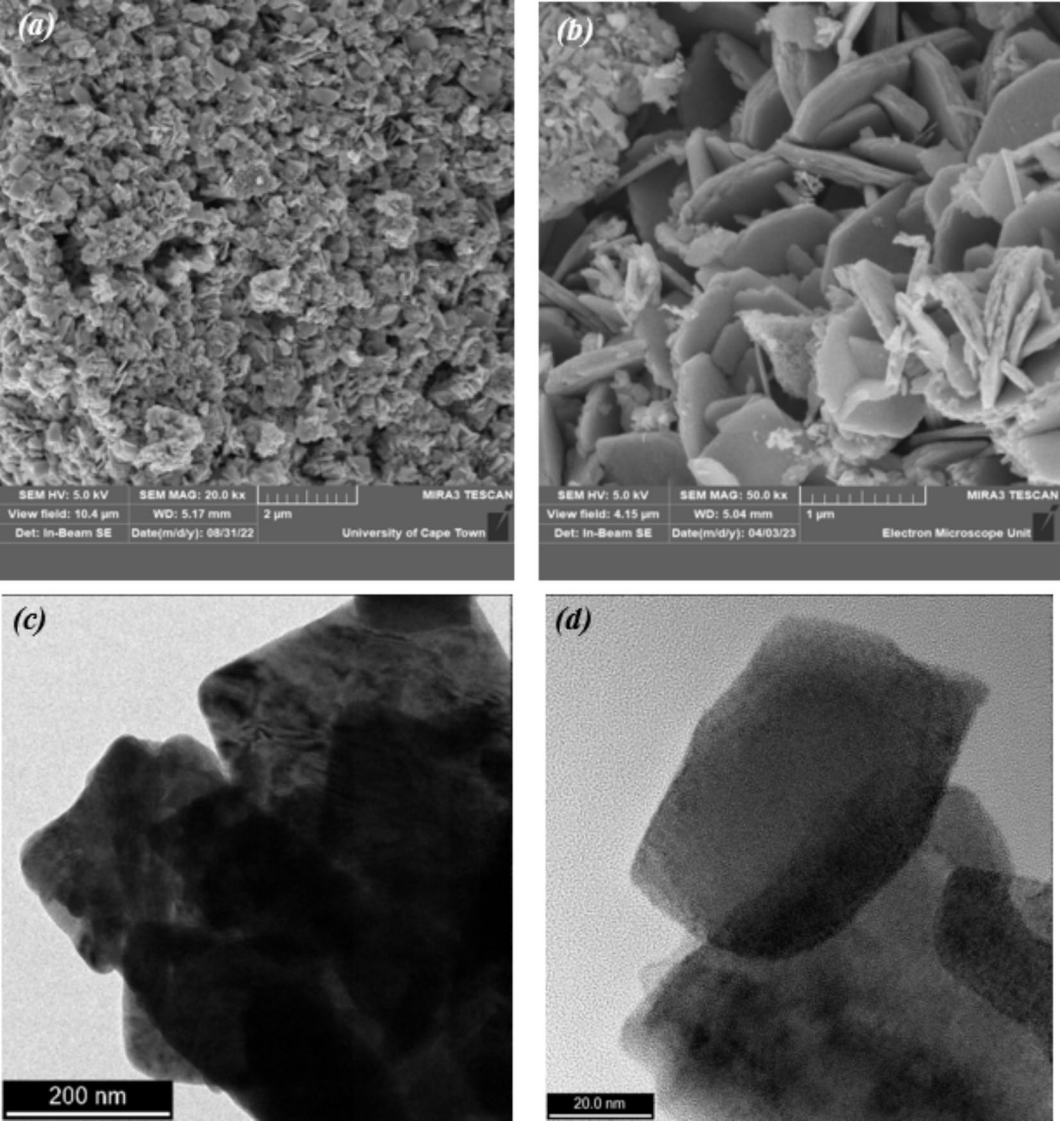
(**a**) Low magnification, and (**b**) high magnification SEM images of WO_3_ nanoflakes. (**c**,** d**) TEM images of WO_3_ nanoflakes.

Figure 4a, b presents the FESEM images of biosynthesized WO_3_. Figure 4a illustrates the overall morphology of WO_3_ and reveals a substantial number of nanoflakes. Figure 4b provides a closer view at higher magnification, revealing a hierarchical structure composed of numerous 2D nanoflakes as the fundamental units. The thickness of these nanoflakes was determined to be approximately 85 nm. The FESEM investigation suggests that spaces between interwoven nanosheets may cause the mesopores in the sample. The WO_3_ microstructure was examined using TEM, and the images are shown in (Fig. 4c,d). Microstructure analysis of the annealed WO_3_ revealed a square sheet approximately 256 nm by 234 nm.

### Optical property

**Fig. 5:**
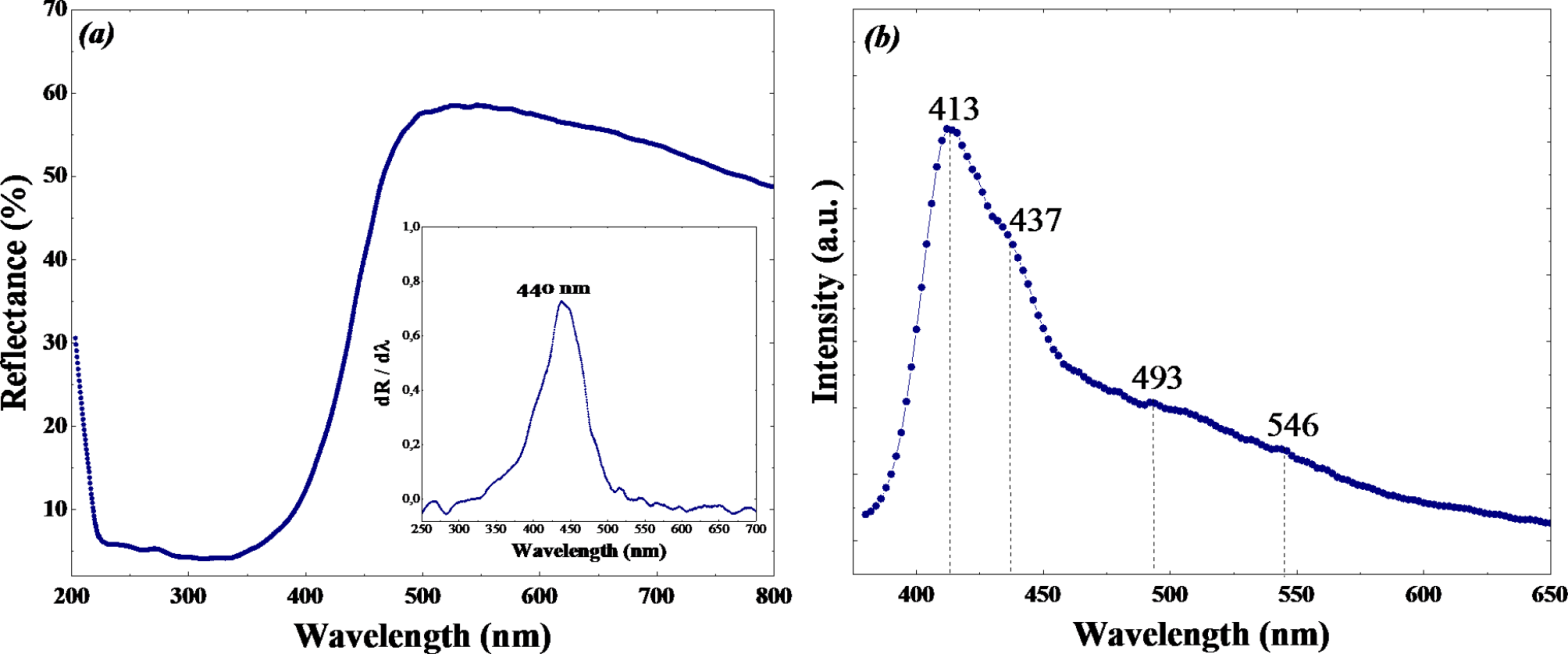
(**a**) UV–vis diffuse reflectance spectra, The Inset shows the optical band-gap energy Eg of WO_3_ adsorbent. (**b**) Fluorescence emission spectrum of WO_3_ adsorbent.

Figure 5a presents the results from diffuse reflectance spectroscopy for WO_3_. The reflectance spectrum resembles that of WO_3_ obtained through various synthesis methods, with the notable difference being a reduction in reflectance above the absorption edge^45^. This decrease in reflectance indicates the formation of defects in the WO_3_ structure. The band gap of WO_3_ were calculated using diffuse reflectance spectroscopy and the Kubelka-Munk method^46^. The inset of Fig. 5a shows the first-order derivative (dR⁄dλ against λ) spectra of WO_3_, which exhibits a single peak at 440 nm. The band gap of WO_3_ was calculated to be approximately 2.8 eV. This result suggests that the WO_3_ nanoflakes are appropriate for visible light absorption.

Figure 5b displays the photoluminescence spectra of the WO_3_ adsorbent, measured at room temperature with an excitation wavelength of 340 nm. The resulting peaks correspond to the ultraviolet, green, and red PL emissions and are located at 413 nm, 437 nm, 493 nm, and 546 nm^47^.

### Surface area investigation

**Fig. 6:**
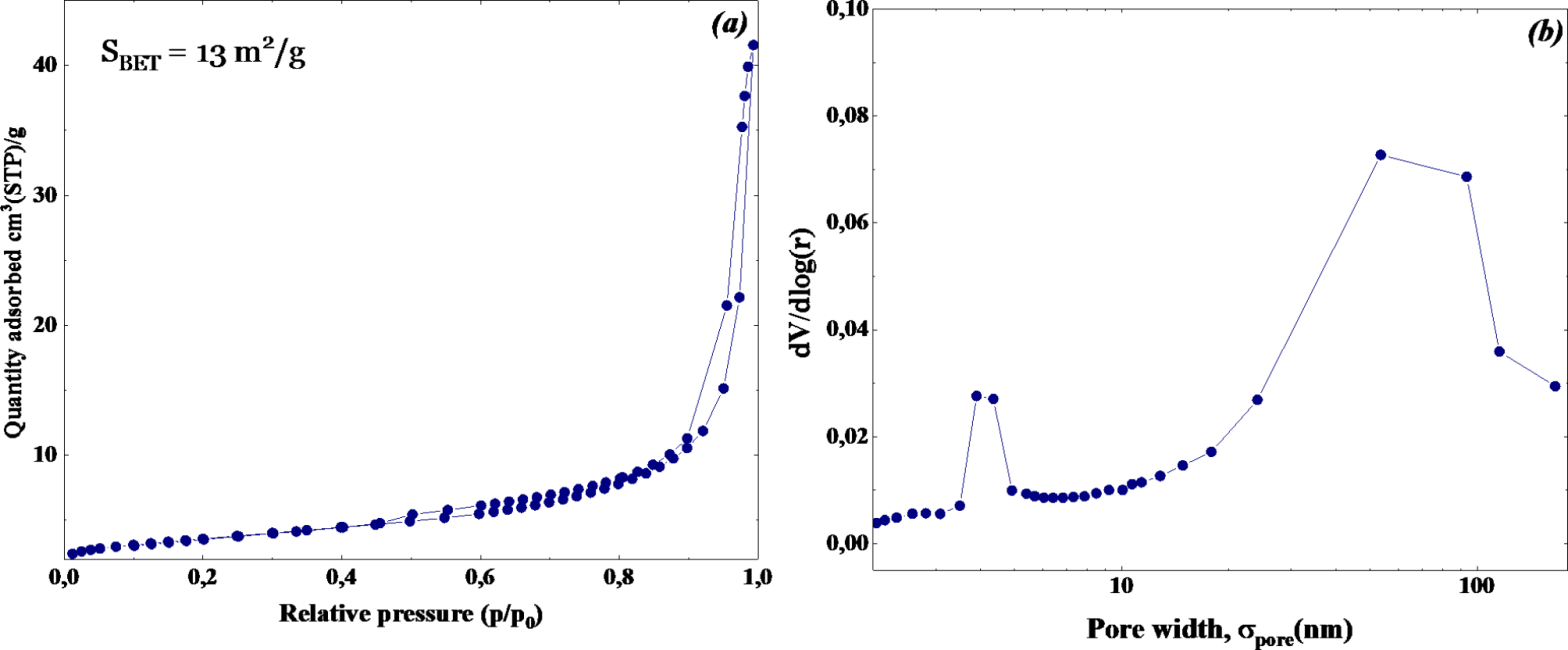
(**a**) N_2_ adsorption-desorption isotherm, and (**b**) The pore size distributions (dV/dlog (r) pore volume vs. pore width σ_pore) for WO_3_ adsorbent.

N_2_ adsorption-desorption experiments at 77 K were used to examine the specific surface area and porosity of WO_3_ nanoflakes. Figure 6a shows the acquired results. According to the International Union of Pure and Applied Chemistry (IUPAC) classification system, the hysteresis loop of WO_3_ shows a type-H3 form, indicative of slit-shaped pores^48^. The WO_3_ BJH desorption pore size distribution, shown in Fig. 6b, exhibits two wide peaks, centered at 4 and 60 nm, and a wide range of pore sizes, from 2 to 100 nm. Large mesopores and macrospores are so clearly present. The WO_3_ nanoflakes demonstrate a specific surface area (SBET) of 13 m^2^/g with a desorption average pore diameter of 17.2 nm and a BJH desorption average pore width of 19.3 nm.

## Dye adsorption study

### Theory

#### Adsorption amount calculation

The adsorbed amount of pollutant dye was calculated using the following mass balance Equation. ^49,50^,

2 $$q_{t} = \frac{{(C_{0} - C_{t} ) \times \:V}}{W}$$

Where $\:{q}_{t}$ and $\:{C}_{t}\:$ are the adsorption capacity per gram of dry weight of the adsorbent ($\:mg.{g}^{-1}$), and the concentration of dye in the solution at time (t), respectively. $\:{C}_{0}$ is the initial concentration of dye in the solution. $\:V$ is the volume of the solution, and $\:W$ is the dry weight of the used $\:{\text{W}\text{O}}_{3}$ (g).

The dye removal efficiency is determined by the following formula,

3 $$\:{Adsorption\:\%}_{\:}=\frac{{(C}_{0}-{C}_{t})}{{C}_{0}}\times\:100$$

The equilibrium adsorption capacity per gram of dry weight of the adsorbate ($\:{q}_{e}$ ($\:mg.{g}^{-1}$)) onto the adsorbent was calculated based on Eq. (4),

4 $$\:{q}_{e}=\frac{{(C}_{0}-{C}_{eq})\times\:V}{W}$$

$\:{C}_{eq}$ is the final or the equilibrium concentration of dye in the solution.

#### Adsorption kinetic study

Kinetic models provide an overview of the adsorption process and can be utilized to acquire a quantitative understanding of it. According to Lagergren^51^, the pseudo-first-order model describes the kinetics in a liquid-solid system in terms of solid capacity. However, the pseudo-second-order model assumes that adsorption obeys the second-order law of chemical sorption^52^. The Eqs. (5) and (6) present the linearized forms of pseudo-first order and pseudo-second order models, respectively.

5 $$\:{ln(q}_{e}-\:{q}_{t})={lnq}_{e}-\frac{{K}_{1}}{2.303}t$$

6 $$\:\frac{t}{{q}_{t}}=\frac{1}{{K}_{2}{q}_{e}^{2}}+\:\frac{t}{{q}_{e}}$$

7 $$\:{h}_{0}={K}_{2}\:{q}_{e}^{2}$$

$\:{q}_{e}$ and $\:{q}_{t}$ represent the amount of organic dye adsorbed on $\:{WO}_{3}$ ($\:{mg.g}^{-1}$) at equilibrium and at specific time $\:{t}_{\:}\left(min\right)$, respectively. $\:{K}_{1}$ and $\:{K}_{2}$ are the first-order and second-order rate constant ($\:{min}^{-1}$), respectively. $\:{h}_{0}$ is the initial adsorption rate $\:(mg.{g}^{-1}.{min}^{-1})$.

#### Intra-particle diffusion study

Weber and Morris proposed a model based on intra-particle diffusion to determine the adsorption mechanisms of the system. Based on this model, the amount taken by most adsorption processes is almost proportional to $\:{t}^{1/2}$, as shown in the following Equation^53^,

8 $$\:{q}_{t}=\:{K}_{id}{t}^{1/2}+I$$

$$\:{K}_{id}$$$$\:(mmol.{g}^{-1}.{min}^{1/2})$$ and $$\:I\:(mmol.{g}^{-1})$$ are the intra-particle diffusion rate constant and the intercept, respectively.

#### Adsorption isotherms study

Adsorption isotherm models are used to evaluate adsorption at the solid-liquid interface. Langmuir^54^ and Freundlich^55^ isotherm models are used to analyze the experimental data and understand the adsorption mechanism of MB onto the WO_3_ nanoflakes. A maximum adsorption capacity for the adsorbent can be predicted using these models. Langmuir and Freundlich models can be expressed using the following equations, respectively:

9 $$\:\frac{{C}_{e}}{{q}_{e}}=\frac{{C}_{e}}{{q}_{max}}+\:\frac{t}{{{K}_{L}q}_{max}}$$

10 $$\:{ln\:q}_{e}={\text{ln}\:{K}_{f}}_{\:}-\frac{1}{n}{\text{ln}\:{C}_{e}}$$

where $\:{C}_{e}$ and $\:{q}_{e}$ are the equilibrium concentration and the amount of MB adsorbed at equilibrium (mg/g), respectively. $\:{q}_{max}$ is the theoretical maximum adsorption capacity (mg/g) and $\:{K}_{L}\:$(L/mg) is the Langmuir equilibrium adsorption constant. $\:{K}_{f}(\text{m}\text{g}/\text{g})\:$ and n are the Freundlich adsorption isotherm constant and the heterogeneity factor, respectively.

### Results and discussions

#### Effect of adsorbent dose and adsorption kinetic study

**Fig. 7:**
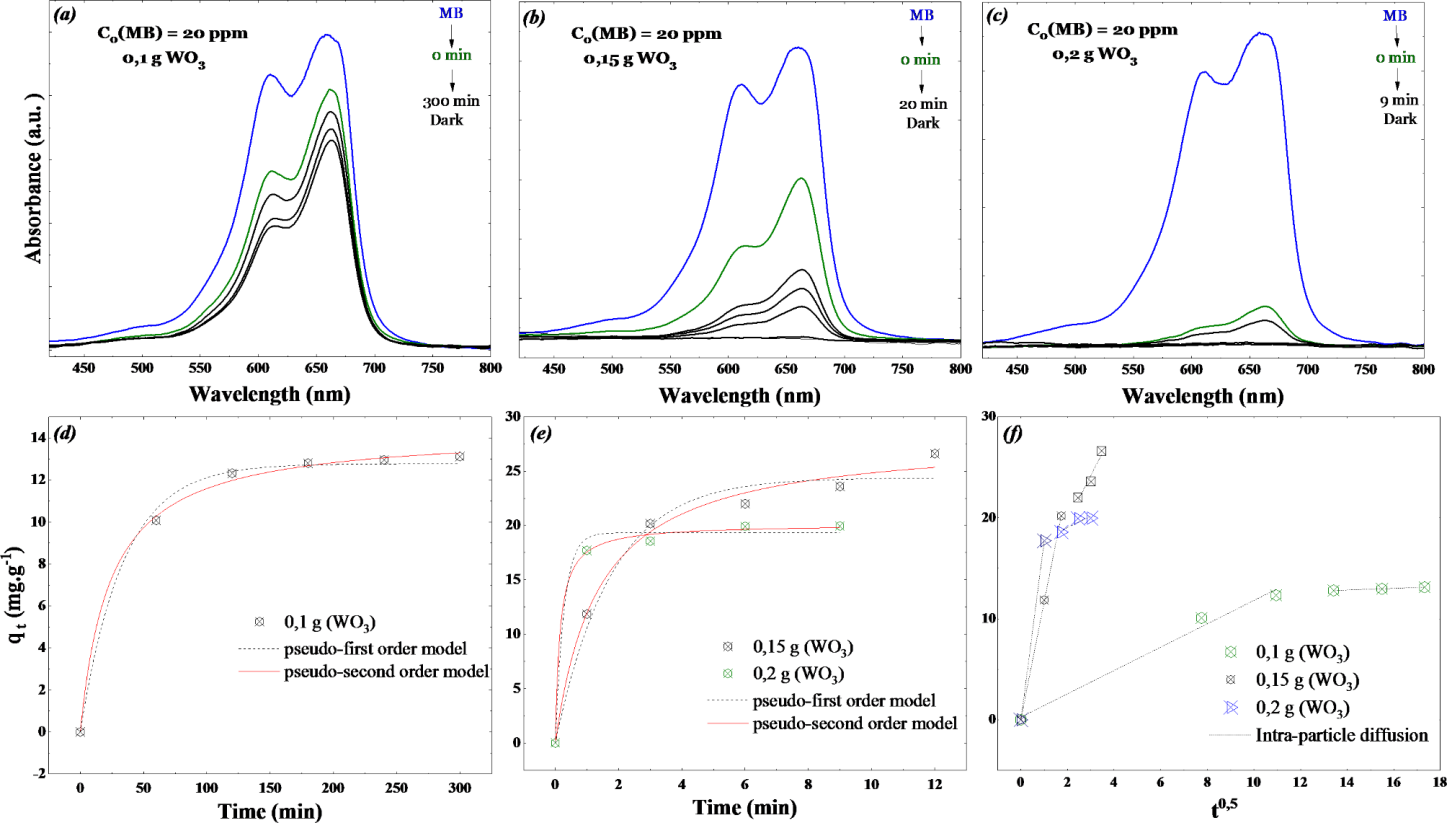
Effect of WO_3_ adsorbent dose on the adsorption efficiency of MB ($\:20\:ppm$) (**a**) 0.1 g, (**b**) 0.15 g, and (**c**) 0.2 g. Experimental results of the adsorption capacity of MB ($\:20\:ppm$) fitted with pseudo-first-order and pseudo-second order kinetics models with the presence of (**d**) 0.1 g, (**e**) 0.15  and 0.2 g of the adsorbent WO_3_ nanoflakes. (**f**) The intraparticle diffusion modeling of the experimental.

A 20 ppm MB solution was used to study how the dose of WO_3_ adsorbent affects the adsorption of methylene blue. The variations in methylene blue solution concentration as a function of time in contact with different doses of the WO_3_ adsorbent (0.1, 0.15, and 0.2 g) are illustrated in (Fig. 7a–c). These results show that the adsorption of the dye at 0 min increases with a higher adsorbent dose. The adsorption of MB increased from 32 to 99.8% for the range of adsorbent doses between 0.1 g and 0.2 g. In addition, the removal of MB dye does not significantly change after an adsorbent dose of 0.15 g / 200 ml. This negligible change could be caused by the overlap of active adsorption sites at doses higher than 0.15 g (WO_3_) /200 ml (20 ppm MB). The adsorption capacities $\:{q}_{t}$ vs. time $\:t$ of different adsorbent doses are illustrated in (Fig. 7d,e). The maximum amount of MB adsorbed at equilibrium is found to be 26.61 mg/g for 0.15 g of adsorbent.

Figure 7d,e displays the pseudo-first and pseudo-second-order models fitted to the experimental data. Table 1 summarizes the fitting results and parameters of the adsorption kinetics models. The pseudo-first-order model showed poor correlation, with a correlation coefficient (R^2^) less than 0.92. In contrast, the pseudo-second-order analysis resulted in fitting regression coefficients of 0.99 for all doses. The pseudo-second-order rate model fits the experimental kinetic data for MB adsorption on WO_3_ nanoflakes better than the pseudo-first-order rate model. In the pseudo-second-order model, the rate-limiting step is not due to the resistance of the boundary layer. Instead, the rate-controlling phase is chemical adsorption involving valence forces, where MB molecules exchange electrons with the WO_3_ adsorbent. This suggests that the adsorption mechanism cannot be adequately explained by an external resistance Equation^22,48^. These results indicate that the adsorption process is likely a chemisorption process. Similar chemisorption behavior has also been observed in the adsorption of MB onto other materials, such as Fe-Mn binary oxide and other metal oxide adsorbents^46^.

**Table 1:** Kinetic parameters of the pseudo-first-order and pseudo-second-order models for the adsorption of MB (20 ppm) onto WO_3_.

		Pseudo-first-order		Pseudo-second-order		Intra-particle diffusion		
Adsorbent dose (g)	$\:{q}_{exp}$ (mg/g)	$\:{q}_{max}$ (mg/g)	*R* ^2^	$\:{q}_{max}$ (mg/g)	*R* ^2^	*K*_*1d*_	*I*_*1*_	*R* ^2^
0.1	13,12	12.77	0.97	14.35	0.99	1.15	0.25	0.97
						0.08	11.69	0.99
0.15	26,61	24.37	0.98	28.12	0.99	11.65	0.061	0.99
						4.49	10.70	0.89
0.2	19,95	19.32	0.92	20.11	0.99	17.71	1.25	0.99
						1.13	16.77	0.66

#### Intra-particle diffusion study

Figure 7f shows the intraparticle diffusion modeling of the experimental data. Table 1 summarizes the results obtained from the simulation. The plots Q_t_ versus t^0.5^ consist of two linear components, indicating that the adsorption process occurs in two stages. The first slope represents the adsorption on the external surface, indicating that a substantial amount of MB adsorbs onto the surface of WO_3_ during this stage. The second slope reflects the boundary layer and intra-particle diffusion. The intercept values for adsorbent dosages of 0,1, 0,15, and 0,2 g were estimated as 10,7, 11,69, and 16,77 mg/g, respectively. Therefore, both liquid membrane transport and intra-particle diffusion control the entire dye adsorption process. Previous research confirms that the two-stage intra-particle diffusion model is a suitable method for describing the adsorption kinetics of this process^20,46^.

#### Effect of pH on adsorption and electrokinetic study

**Fig. 8:**
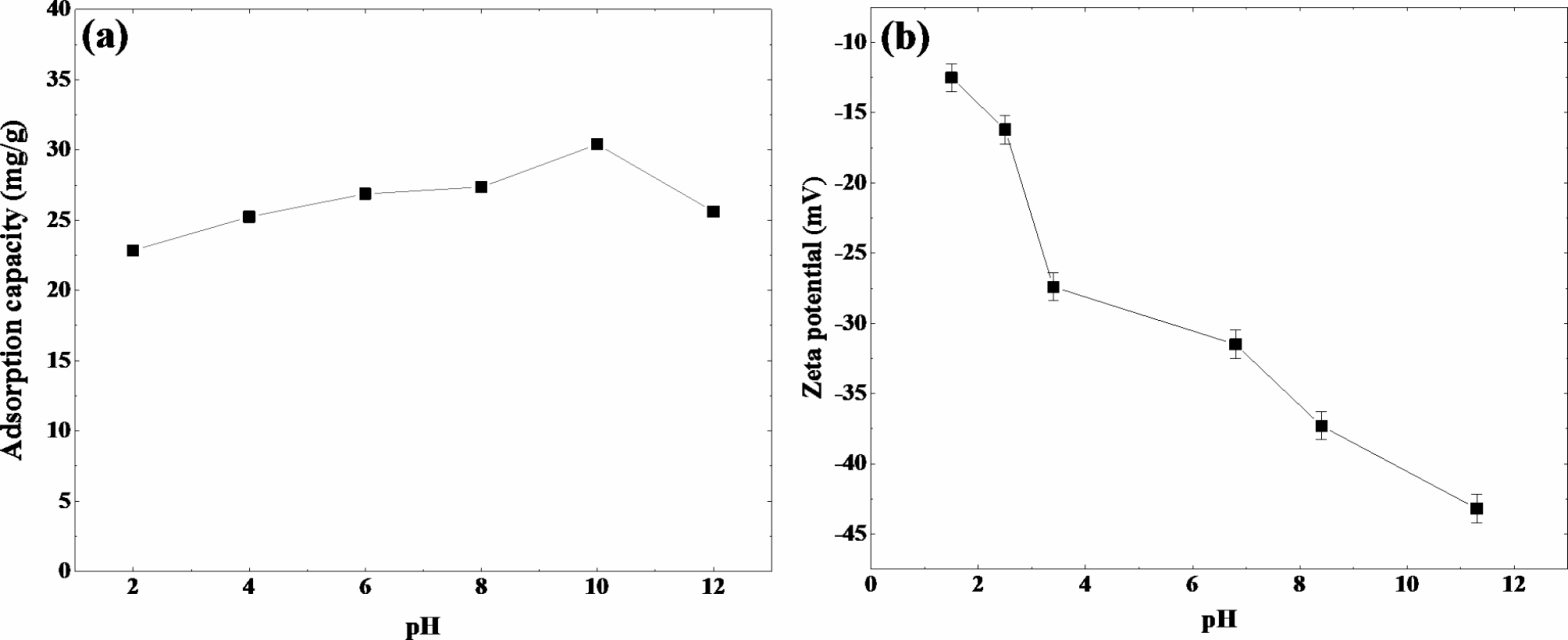
(**a**) Effect of solution pH on the adsorption capacity of the WO_3_ nanoflakes (**b**) zeta potential vs. pH curves of WO_3_ nanoflakes measured in the pH 1.5–11 range.

The pH value plays a crucial role in adsorption processes, as variations in pH can influence the ionization of functional groups in both the adsorbent and adsorbate, thereby affecting their interaction. The effect of pH on the adsorption capacity of WO_3_ nanoflakes toward MB was investigated across a pH range of 2 to 12, as shown in (Fig. 8a). The results demonstrate that the adsorption capacity of WO_3_ gradually increases from pH 2 to 10, reaching an optimal point, and then rapidly decreases when the pH exceeds 10. At low pH values (around pH 2), the solution is highly acidic, leading to an excess of hydrogen ions (H⁺). These H⁺ ions compete with the positively charged MB molecules for available adsorption sites on the surface of WO_3_ nanoflakes, resulting in lower adsorption capacity. The competition between H⁺ and MB at these sites explains the reduced adsorption in highly acidic conditions. In contrast, at higher pH values (above pH 10), the adsorption capacity declines sharply due to the presence of excess hydroxide ions (OH⁻). The OH⁻ ions may adsorb onto the surfaces of both MB and WO_3_ nanoflakes, causing electrostatic repulsion between the negatively charged surfaces. This repulsion reduces the equilibrium adsorption uptake of MB, thereby decreasing the adsorption efficiency under alkaline conditions. Thus, the adsorption process is most efficient in the near-neutral to slightly alkaline pH range, where electrostatic attraction between the positively charged MB and the negatively charged WO_3_ surface is maximized.

Figure S1 and Fig. S2 show the zeta potential and the size distribution spectra of the biosynthesized WO_3_ dispersed in dionized water at natural pH, respectively. The obtained results are presented in Table 2. The biosynthesized WO_3_ exhibits a zeta potential of -31.5 mV at natural pH; this highly negative value indicates its strongly anionic nature and confirms that the surface of WO_3_ is highly negatively charged. The average hydrodynamic size of particles for WO_3_ dispersed in dionized water was obtained to be around 510 nm. The table shows as well the parameter P_dl_, which is the polydispersity index (PDI) multiplied by the intensity-weighted mean diameter (dl). The PDI reflects the spread of particle sizes, with lower values indicating uniform sizes and higher values indicating more variation. The combined parameter PDI×dl captures both the average particle size and the variability in size within the sample. The WO_3_ showed a P_dl_ value of 0,269, indicating a homogeneous and narrow size distribution, where the majority of particles were of similar size. This uniformity in particle size distribution suggested a well-controlled synthesis process.

**Table 2:** Zeta potential and hydrodynamic size of WO_3_.

Sample	Zeta potential (mV)	Size (nm)	*P*_dI_	Conductivity (mS/cm)
WO_3_	-31,5	510,0	0,27	1,10

Figure 8b shows the zeta potential vs. pH curves of WO_3_ nanoflakes measured in the pH 1.5–11 range. As can be seen, the overall zeta potential decreased as the pH increased and shows negative values in the whole pH range. Such behavior was obtained in previous studies, and it is related to the particle shape and to the high dispersity of the nanoflakes^56,57^. This indicates that the surface of WO_3_ nanoflakes possessed a dominant negative charge. In contrast, MB’surface was positively charged. Thus, MB molecules easily attach to the negatively charged sites of WO_3_ nanoflakes due to electrostatic attraction. Therefore, it can be concluded that electrostatic attraction likely plays a key role in the adsorption of MB onto WO_3_ nanoflakes.

#### Adsorption isotherm study

**Fig. 9:**
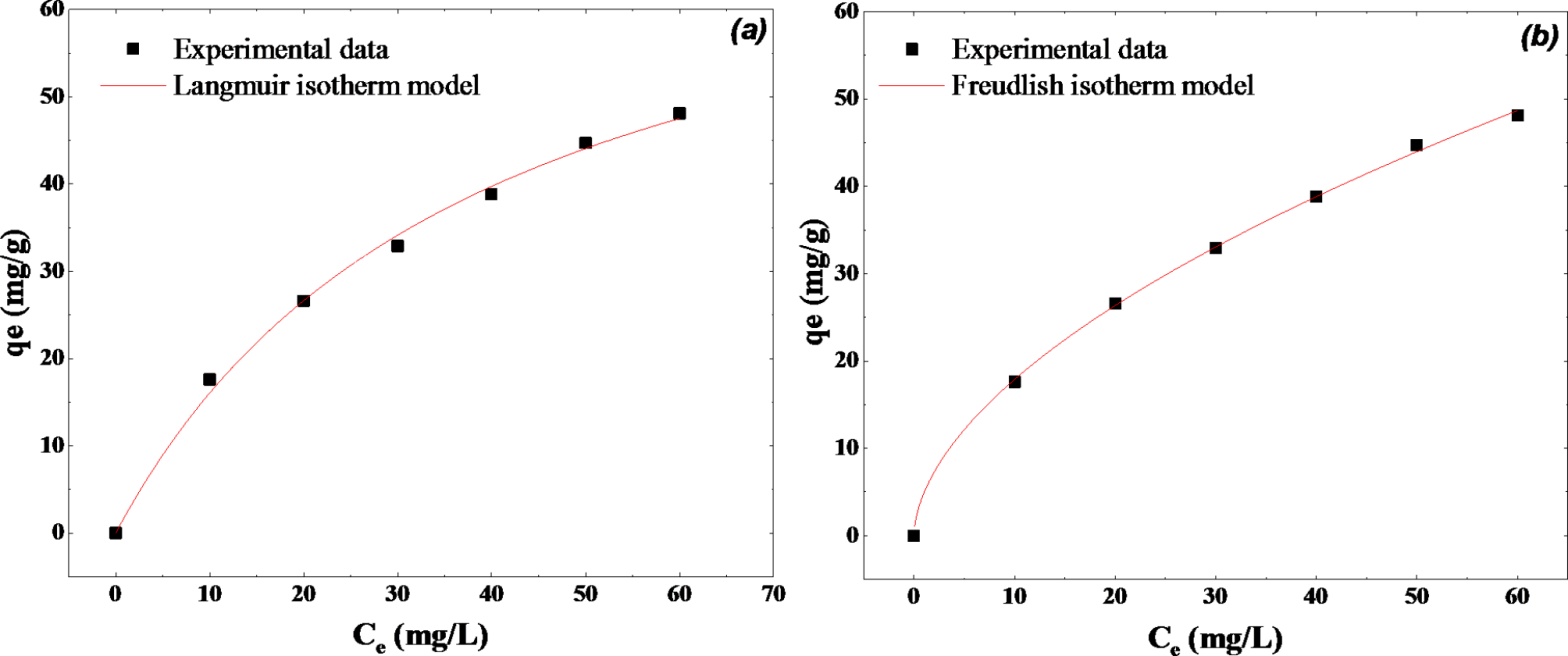
Adsorption isotherm for MB on WO_3_ (0,15 g).

The MB adsorption isotherm experiments were conducted using initial concentrations between 10 and 60 mg/L. The adsorption time was 24 h, which is sufficient to reach equilibrium. Figure 8 shows that the adsorption capacity of WO_3_ increased with increasing concentrations of MB until it gradually reached saturation. Figure 9a and Fig. 9b illustrate the fitting of experimental data for the Langmuir and Freundlich isotherm models, respectively. According to Table 3, both the Langmuir and Freundlich models fit the experimental data simultaneously (*R* = 0.99). It may have both single-layer and multi-layer characteristics in series, indicating that the adsorption process in this system might be complex. The Langmuir model suggests that adsorption site energy remains constant, regardless of the surface coverage. This supports the obtained results of adsorption kinetic studies, as adsorption occurred at even spots on the WO_3_ surface. Once a dye molecule adsorbs onto a site, no more dye molecules can adsorb at that site^58^. According to the fitting parameters, the maximum adsorption capacity of WO_3_ for MB approximately 78.14 mg/g. This suggests that the nanoflake shape improves the adsorption capacity of WO_3_, leading to better results compared to other shapes of WO_3_ prepared using other methods^19,20,59–65^. A summary of the results obtained in other studies is presented in Table 4. As can be seen from Table 4, our adsorption capacity is comparable to a few studies and better than numerous literature reports. Although other studies report higher adsorption capacities, the materials used in those studies differ in properties such as amorphous nature, crystal phase, and hydrates. The adsorption of dye pollutants is strongly affected by the material’s structure, crystallinity, and hydration.

The value of the separation factor $\:{K}_{L}$ lies between 0 and 1, which means the adsorption process is favorable. As the value is 0.025 L/mg (very close to 0), this indicates a more favorable adsorption condition of the MB onto WO_3_ nanoflakes. The Freundlich model is empirical and describes how adsorption occurs on uneven surfaces. This model is more effective in explaining multilayer adsorption, involving interactions such as physical adsorption with weak van der Waals forces. This model accounts for adsorption on heterogeneous surfaces^55^. Since the Freundlich model fits our experimental results well, it suggests that physical adsorption and weak van der Waals forces are involved in the adsorption of MB on WO_3_ nanoflakes. Table 5 summarizes the adsorption capacities of various metal oxides toward methylene blue. The results indicate that manganese dioxide nanoparticles exhibit the highest adsorption capacity among the studied metal oxide nanoparticles. However, the table also shows that studies on the same materials, such as MnO₂ and V₂O₅, yield different results. This variability clearly demonstrates that adsorption capacity is influenced by several factors, including particle shape, size, surface porosity, hydration, and crystallinity. The adsorption capacity of methylene blue obtained in this work is comparable to the results reported for other metal oxides, further underscoring the material’s effectiveness in this context.

**Table 3:** Isotherm adsorption analysis parameters for removal of methylene blue by WO_3_ nanoflakes.

Langmuir constants	$\:{q}_{max}$ (mg/g)	$\:{K}_{L}$ (L/mg)	*R* ^2^
	78.14	0.025	0.99
*Freundlich constants*	$\:{K}_{F}$ (mg^1 − 1/*n*^ L^1/*n*^ g^− 1^)	$\:{n}_{f}$	*R* ^2^
	4.94	1.67	0.99

**Table 4:** Methylene blue adsorption capacity of different WO_3_ nanostructures from various studies.

Adsorbent	Synthesis method	Adsorption capacity (mg/g)	Ref
WO_3_ (Crystalline)	Combustion	8	^21^
WO_3_ (Crystalline)	Commercial	12.3	^59^
WO_3_ (Amorphous)	Thermal deposition	600	^60^
WO_3_ (Crystalline)	Hydrothermal	73.0	65
WO_3_ (amorphous)	glancing angle deposition	149.8	^62^
WO_3_ (hydrates)	Ion exchange	247.3	63
WO_3_ (Crystalline)	hydrothermal	57.6	^64^
WO_3_ (Crystalline)	Acid precipitation	32.4	^65^
WO_3_ (Crystalline)	Solution-precipitation method	57.7	^20^
WO_3_ (Crystalline)	Green synthesis	78.14	Present work

**Table 5:** Methylene blue adsorption capacity of different metal oxides from various studies.

Adsorbent	Adsorption condition	Adsorption capacity (mg/g)	Ref
CuO NPs	Adsorbent dosage = 5 mg/L Contact time = 10 min Initiat adsorbate concentration = 20 mg/L	61	^66^
MnO_2_ NPs	Adsorbent dosage = 10 mg/L Contact time = 10 min Initiat adsorbate concentration = 10 mg/L	627.1	^67^
MnO_2_ NPs	Adsorbent dosage = 20 mg/L Contact time = 60 min Initiat adsorbate concentration = 20 mg/L	22.2	^68^
Fe_3_O_4_ NPs	Adsorbent dosage = 1 g/L Initiat adsorbate concentration = 8.5 mg/L	7.25	^69^
V_2_O_5_ NPs	Adsorbent dosage = 150 g/L Initiat adsorbate concentration = 25 mg/L	400	^70^
V_2_O_5_ Nanorods	Adsorbent dosage = 0.5 g/L Initiat adsorbate concentration = 230 mg/L	149.8	^71^
TiO_2_ nanotubes	Adsorbent dosage = 60 g/L Initiat adsorbate concentration = 20 mg/L	133.3	^72^
WO_3_ (Crystalline)	Adsorbent dosage = 15 g/L Initiat adsorbate concentration = 60 mg/L	78.14	Present work

### Molecular dynamic simulation (MDS) study

There are two forms of adsorption, namely chemisorption and physisorption, determined by how materials interact with pollutants^17,73^. Molecular dynamics (MD) is used in computational studies for wastewater treatment to understand the behaviors and interactions of pollutants or solvents and the adsorbent^74^. Examining the interaction between methylene blue and WO_3_ through computational analysis enables a better understanding of how this dye is adsorbed and decomposed. This investigation holds the potential to enhance the development of materials for purifying water contaminated with methylene blue.

#### Computational methods

**Fig. 10:**
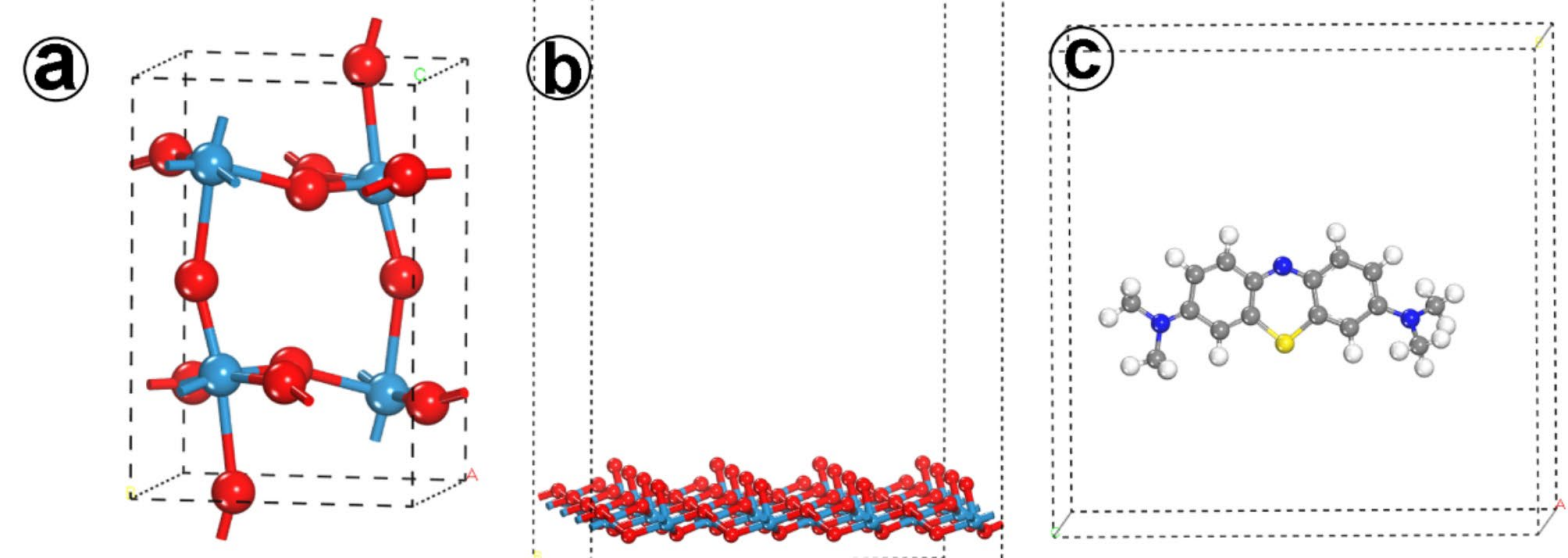
(**a**) Bulk structure of WO_3_, (**b**) The surface (002) of WO_3_, (**c**) Methylene blue molecule.

A theoretical investigation was conducted to explore the interactions of the Methylene blue molecule with the (002) surface of tungsten trioxide (WO_3_) using Materials Studio 7.0 software. The initial structure of bulk WO_3_ shown in Fig. 10a was retrieved from the software’s library and subsequently relaxed to its energetically favorable state. All structural configurations for the adsorbent and adsorbate were optimized employing the “Forcite” Geometry Optimization module, utilizing a smart algorithm for atomic movement in the relaxation process, with convergence criteria set to 2.0e^− 6^ Kcal/mol, 1.0e^− 4^ Kcal/mol, 0.001 GPa, and 1.0e^− 6^ Å for energy, force, stress, and displacement, respectively. The universal forcefield was employed, with electrostatic interactions computed via a summation method, and van der Waals interactions were determined based on a cutoff distance, spline width, and buffer width of 18.5 Å, 1 Å, and 0.5 Å, respectively. To identify the potential adsorption configurations of methylene blue molecule (Fig. 10c) on the WO_3_ (002) surface, shown in Fig. 10b, a systematic exploration of the configurational space of the substrate-adsorbate system was conducted through manual searches.

During the simulated adsorption process, the adsorbate molecules were treated as rigid bodies, permitting adjustments in their positions and orientations. X-ray diffraction (XRD) analysis revealed that the primary adsorption plane was the (002) plane; consequently, the bulk structure of WO_3_ was cleaved, and a 15 Å vacuum layer was introduced to preclude interactions between periodic surface layers. The simulations involved the relaxation of each structure within a P1 periodic crystal, followed by the alignment of each molecule for adsorption on the WO_3_ surface via its functional group. Subsequently, the adsorption energy was calculated once the relaxation met the predefined convergence criteria. The adsorption energy $\:{E}_{ads}$ for each molecular orientation could be calculated via Eq. (11): ^75,76^:

11 $$\:{E}_{ads}={E}_{total}-{E}_{surface}-{E}_{molecule}$$

Where $\:{E}_{total}$ is representing the total energy of the molecule while adsorped on the surface, $\:{E}_{surface}$ is representing the WO_3_ (002) surface without any molecules adsorbed on it, and $\:{E}_{molecule}$ is representing the energy of the Methylene blue alone without adsorption.

#### Results and discussion

**Fig. 11:**
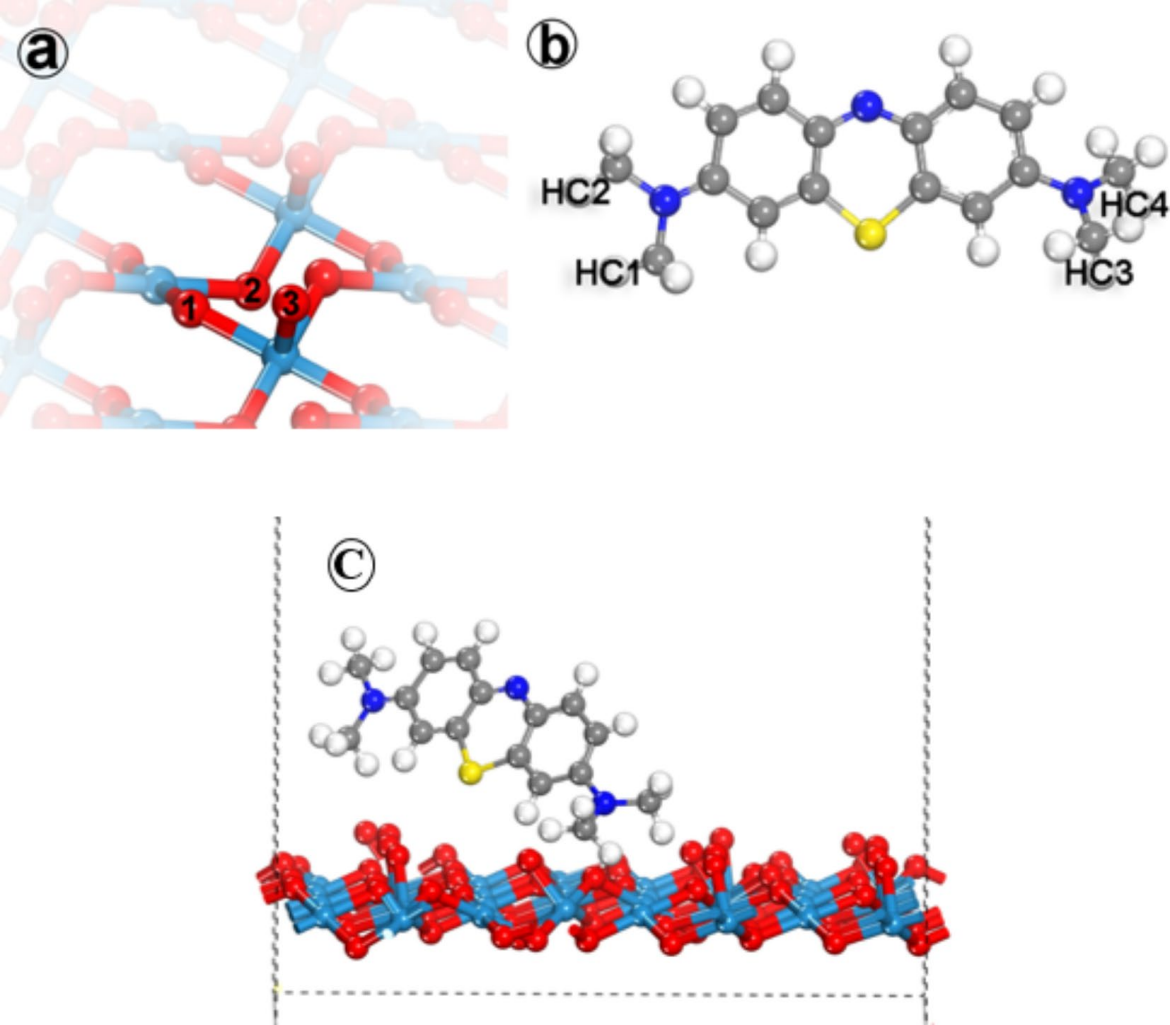
Specified atomic position for (**a**) WO_3_ surface, (**b**) Methylene blue molecule, and (**c**) Adsorption position of MB onto (002) WO_3_ surface.

**Fig. 12:**
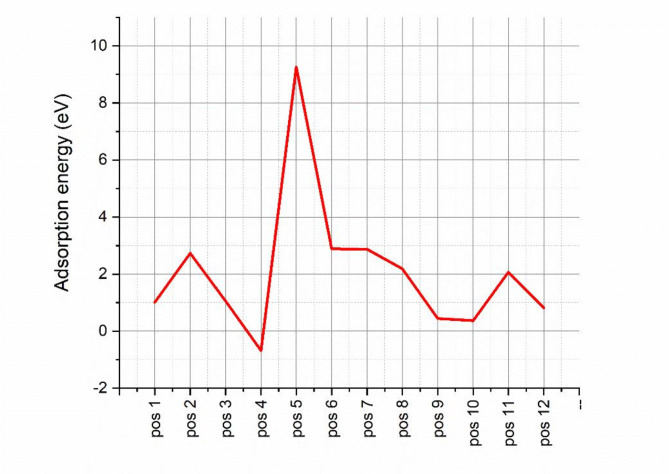
The adsorption energy for each position.

Before setting the possible scenarios for the adsorption of Methylene blue on the WO_3_ surface, the possibility of adsorbing Methylene blue via its central atoms was excluded due to the steric hindrance of its neighboring groups. The total energy of formation for the Methylene blue molecular structure was found to be 2.08 eV, while for the (002) surface of WO_3_, it was 81.17 eV. For simplicity, each adsorbate atom has been given a number, as shown in Fig. 11. The most favorable adsorption position was selected based on its adsorption energy, as shown in (Table 6). Each possible atomic position in both the adsorbent and adsorbate was specified by a number to ease the discussion, as shown in Fig. 11. The adsorption energy showed a fluctuation among all the positions, as shown in Fig. 12, with the least adsorption energy in position number 4. The adsorbent of the WO_3_ surface was specified by different types of oxygen atoms according to their orientation, as O1 has an orientation to the top, O2 has an orientation to the bottom, and O3 has an orientation to the top with dangling bonds. For the methylene blue, there are six possible adsorption sites, as the adsorption could be via one hydrogen atom of the methylene groups or by both hydrogens. The lowest adsorption position was found to be position number four, where there is only one hydrogen atom with a partial positive charge attached to the methylene group attracted to the oxygen number, where there is a partial negative charge on it, resulting in an adsorption energy of -0.68 eV. That means the mechanism of adsorption of the methylene blue molecule over the WO_3_ surface will be started by the electrostatic attraction of the hydrogen atom towards oxygen number two until the bond length between them reaches 1.12 A.

**Table 6:** Formation and adsorption energy calculated via MDS for each adsorption position.

Position number	Adsorption position	Formation Energy (eV)	Adsorption Energy (eV)	Graphical representation
	WO_3_(002) supercell	324.6825704	–	
	WO_3_(002)	81.17007395	–	
Position 1	HC1@O1	84.25551212	1.009211238	Figure S3
Position 2	HC1HC2@O1O1	85.97998078	2.733679905	Figure S4
Position 3	HC1HC2@O1O2	84.30451748	1.058216607	Figure S5
Position 4	HC1@O2	**82.56358282**	**-0.682718053**	Figure S6
Position 5	HC1HC2@O2	92.50303926	9.256738382	Figure S7
Position 6	HC1&HC2@O1	86.13260824	2.886307365	Figure S8
Position 7	HC1&HC2@O2	86.1215089	2.875208025	Figure S9
Position 8	HC1&HC2@O3	85.43741168	2.1911108	Figure S10
Position 9	HC1@O3	83.68734625	0.441045369	Figure S11
Position 10	HC1@O1O3	83.61948482	0.373183946	Figure S12
Position 11	HC1@O2O3	85.31806662	2.071765747	Figure S13
Position 12	HC1HC3@O3O3	84.05832781	0.812026931	Figure S14

### Adsorption mechanism

#### FT-IR before and after adsorption

**Fig. 13:**
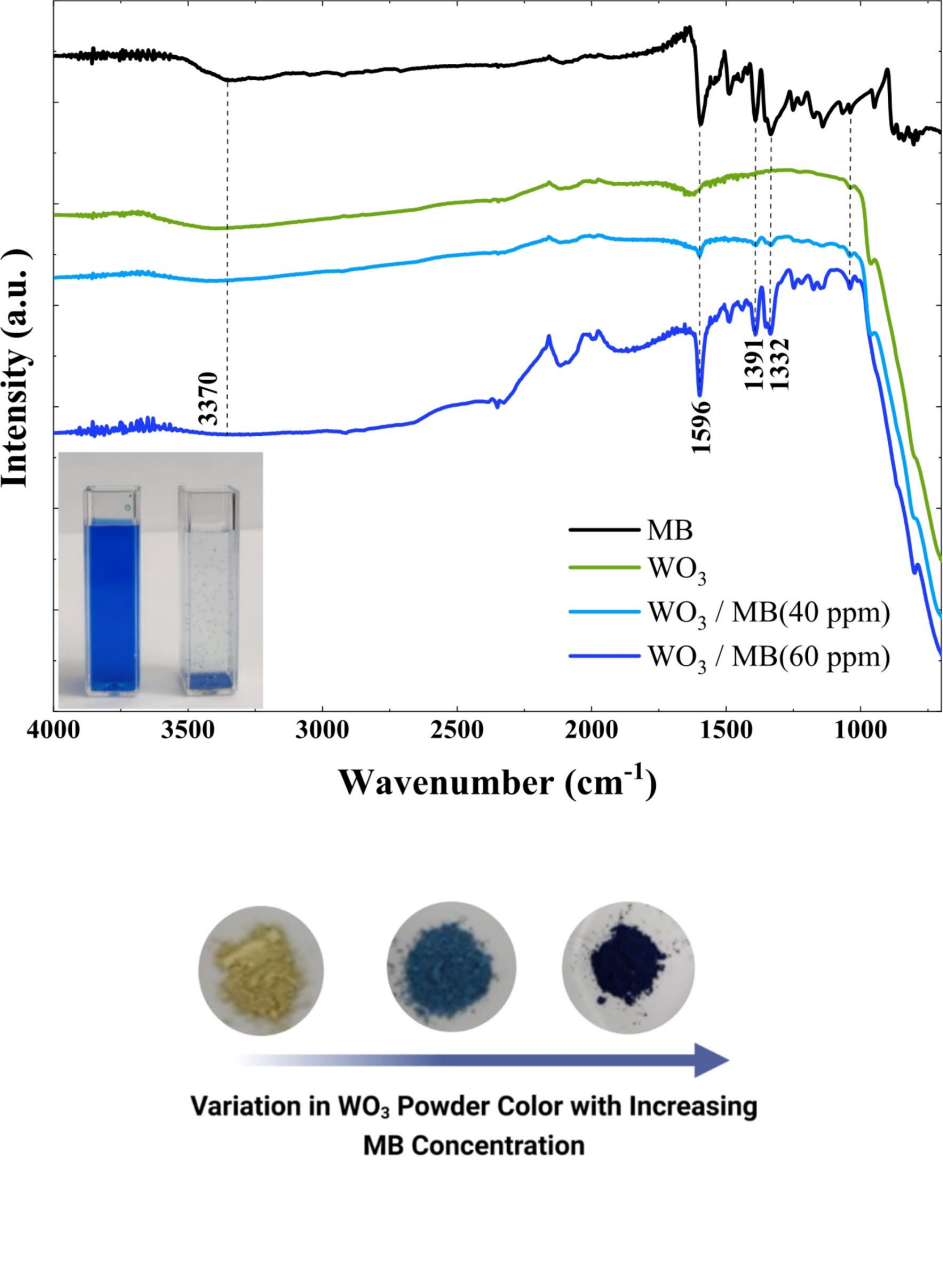
FTIR spectra of methylene blue (MB), WO_3_ before and after MB adsorption. Variation in WO_3_ powder colour with increasing methylene blue concentration.

An FTIR spectral study was performed to investigate the chemical groups on the adsorbent surface before and after adsorption. Figure 13 presents the FTIR spectra of methylene blue (MB), WO_3_ before adsorption (WO_3_), WO_3_ after adsorption of MB-40 ppm (WO_3_/ MB (40 ppm)), and WO_3_ after adsorption of MB-60 ppm (WO_3_/ MB (60 ppm)). As can be seen, the FTIR spectrum of MB revealed distinct vibrational bands corresponding to C-N single bonds, aromatic rings, and CH_3_ groups at 1596, 1391, and 1332 cm^− 1^. The same stretching vibration peaks were observed in the FTIR spectrum of WO_3_/ MB (40 ppm). Moreover, we can notice the absence of the peak at 3370 cm^− 1^ in both adsorbent spectra following the adsorption of MB. This indicates the involvement of NH/OH in the adsorption mechanisms of methylene blue^20^. Similar results have been found by other researchers in related investigations^20,60^. As the methylene blue concentration increased in the solution, an increase in MB peak intensity was observed in the WO_3_/ MB (60 ppm) spectra as opposed to the WO_3_/ MB (40 ppm) spectrum, indicating substantial and physical adsorption of MB onto WO_3_. These findings suggest the adsorbent’s surface saturation at high methylene blue concentrations and can be confirmed by the dark blue color of WO_3_/ MB (60 ppm) powder (Fig. 13). In all concentrations used, MB adsorption onto WO_3_ was successful, and the increase in methylene blue concentration revealed the appearance of a new adsorption mechanism.

#### Adsorption mechanism

**Fig. 14:**
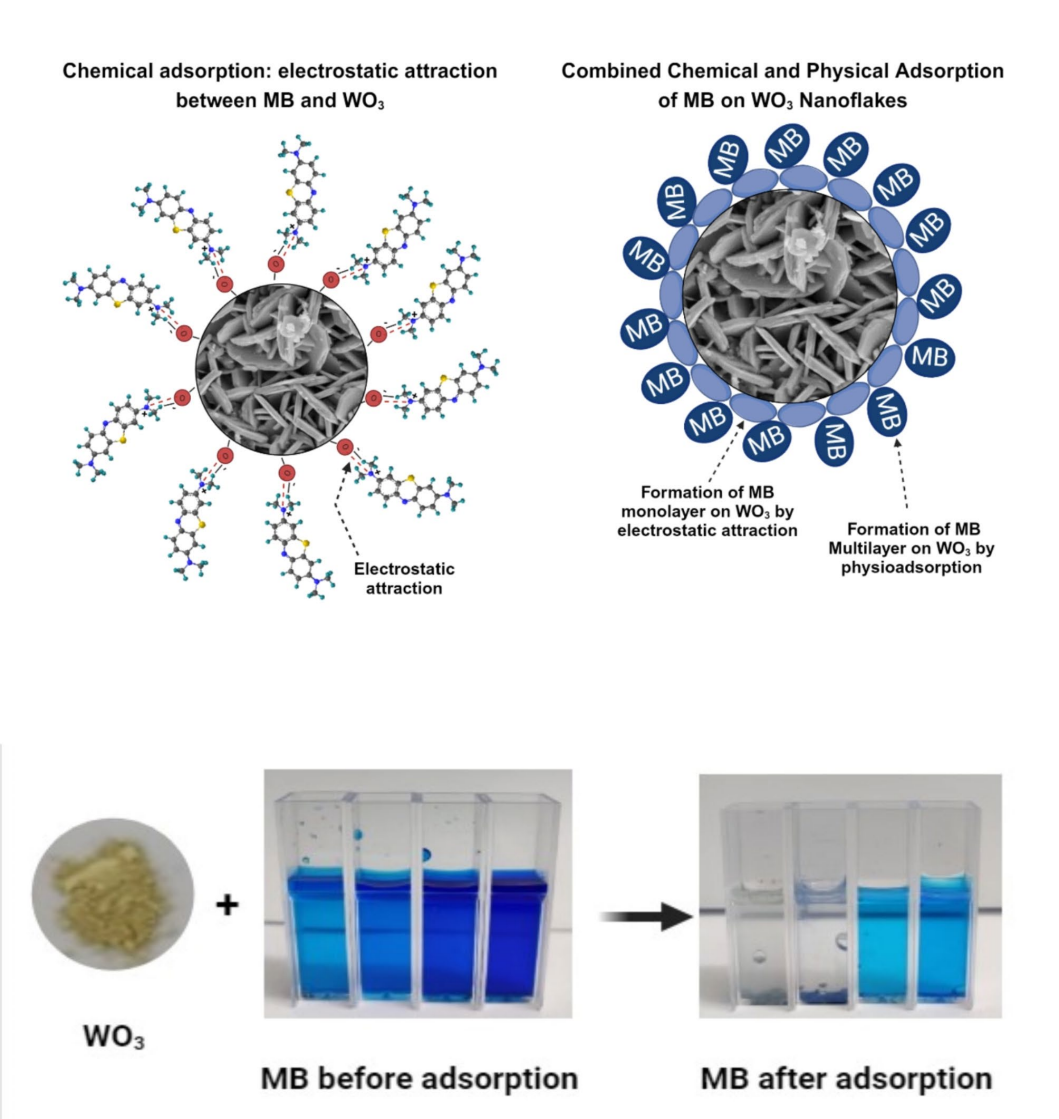
Illustrative diagram and the corresponding mechanism supporting the adsorption of methylene blue onto WO_3_.

This adsorption mechanism is based on results obtained from modeling, molecular dynamic simulations (MDS), FTIR analysis, and zeta potential measurements. All these results consistently support the same conclusion about the adsorption process. Following the adsorption process, no additional peaks or bands were found in the adsorbent’s spectra. These results eliminate the possibility of new chemical entities forming during the adsorption process. Consequently, the hypothesis of the appearance of a new chemical adsorption mechanism can be refuted, while the hypothesis of a new physical adsorption mechanism is supported. The electrokinetic study confirmed that the surface of WO_3_ nanoflakes is highly negatively charged, with a zeta potential of -31.5 mV at natural pH. This strong negative charge indicates a dominant anionic nature on the surface of WO_3_ nanoflakes, which plays a crucial role in the adsorption process. As shown in the inset of Fig. 13, after MB molecules were adsorbed into the porous microstructures of WO_3_ nanoflakes, precipitation occurred at an initial MB concentration of 40 ppm. This phenomenon has been observed before by Jian et al. (2013); it is explained by the self-separation of the adsorbent from water^60^. Numerous studies have proven that the surface of WO_3_ is negatively charged at a natural pH^20^. The adsorbent surface is negatively charged at a neutral pH. Based on the zeta potential, the adsorption isotherm, and the MDS results, it can be assumed that the adsorption of the cationic dye MB at low concentrations on the WO_3_ nanoflakes is due to electrostatic attraction^20,60^. As the concentration of methylene blue increases in the solution, the adsorption mechanism changes. It shifts from electrostatic attraction to a combination of electrostatic attraction and physical adsorption, influenced by the effects of surface saturation. An overview of the adsorption mechanism of MB onto WO_3_ was schematized in (Fig. 14).

### Reusability and regenerative studies

**Fig. 15:**
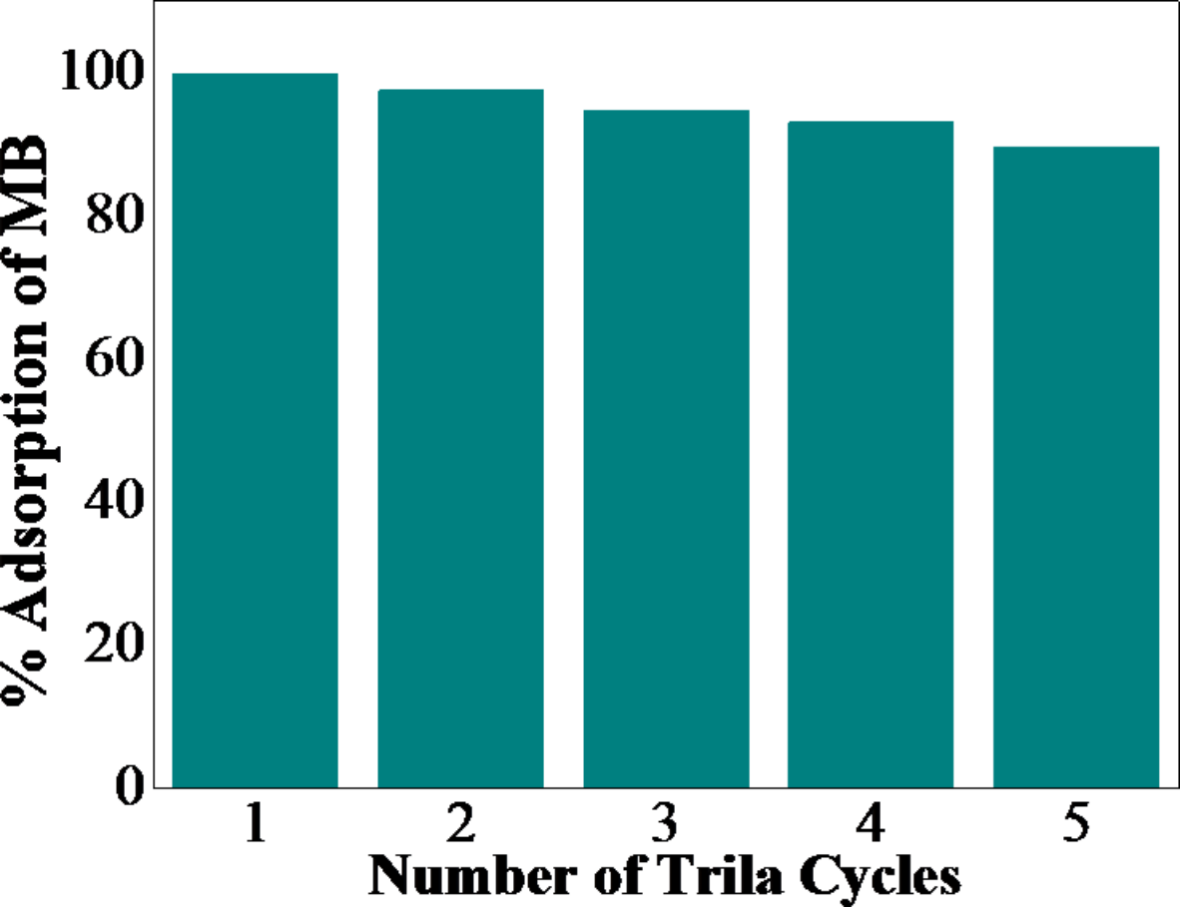
Reusability and regeneration study of WO_3_ adsorbent for adsorption of MB.

In order to make the adsorption process more economical, it is crucial to regenerate and repurpose the adsorbent material. For testing the reusability of WO_3_, the powder was repeatedly regenerated and tested for its adsorption activities for five cycles. Before being tested on the next cycle, the powder was heated at 200 °C for 2 h. Based on the results shown in Fig. 15, it is evident that the adsorption capacity of the material was affected slightly even after five cycles, resulting in a 10% reduction in the removal efficiency of MB. Therefore, WO_3_ catalysts remain stable, reusable, and recyclable for organic dye adsorption.

## Conclusion

In this study, WO_3_ nanoflakes were successfully synthesized using Hyphaene thebaica fruit extract, a green and environmentally friendly route. The synthesized WO_3_ nanoflakes exhibited a high crystallinity and a specific surface area of 13 m^2^/g, with an average pore size of 19.3 nm. These WO_3_ nanoflakes exhibited superior adsorption performance for methylene blue (MB), with a maximum adsorption capacity of 78.14 mg/g. The adsorption kinetics followed a pseudo-second-order model with a correlation coefficient (R^2^) of 0.99, indicating chemisorption as the predominant mechanism. The intraparticle diffusion study confirmed a two-stage adsorption mechanism involving surface adsorption and intraparticle diffusion. Adsorption isotherms fitted both Langmuir and Freundlich models with high correlation coefficients (R² = 0.99), suggesting a complex adsorption process that combines monolayer and multilayer adsorption. Molecular dynamic simulations have provided further insights and revealed an electrostatic attraction between MB and the WO_3_ (002) surface. The most favorable adsorption position has an energy of -0.68 eV. The electrokinetic study confirmed that the WO_3_ nanoflakes have a strongly negative zeta potential of -31.5 mV and a uniform particle size of around 510 nm. The negative charge on WO_3_ supports the electrostatic attraction between WO_3_ and MB, as previously confirmed by adsorption isotherm studies, molecular dynamic simulations, and FTIR analysis. The adsorption mechanism was determined from modeling, MDS, FTIR, and zeta potential measurements. These results show that the interaction between WO_3_ and methylene blue is primarily due to electrostatic attraction. At higher MB concentrations, the physical adsorption played a role due to surface saturation. The reusability tests showed that the WO_3_ nanoflakes retained 90% of their adsorption efficiency after five cycles, confirming their stability and potential for practical applications.

## Electronic supplementary material

Below is the link to the electronic supplementary material.

Supplementary Material 1

## Data Availability

The datasets used and/or analysed during the current study available from the corresponding author on reasonable request.
