# Supplementary material for: Experimental and theoretical insights into the adsorption mechanism of methylene blue on the (002) WO3 surface
Source: Sci Rep. 2024 Nov 6;14:26991. doi: 10.1038/s41598-024-78491-3 (PMC11541561; doi:10.1038/s41598-024-78491-3)
Supplement: Supplementary file 1 — Supplementary Material 1 [file 41598_2024_78491_MOESM1_ESM.docx]

**Supporting Information**

Experimental and Theoretical Insights into the Adsorption Mechanism of Methylene Blue on the (002) WO_3_ Surface

Khaoula Hkiri^1^, Hamza Elsayed Ahmed Mohamed^1^, Mohamed Mahrous Abodouh^2,3^, and Malik Maaza^1^

*^1^ UNESCO UNISA Africa Chair in Nanoscience and Nanotechnology, College of Graduate studies, University of South Africa, South Africa.*

*^2^Energy Materials Laboratory, Physics Department, School of Sciences and Engineering, The American University in Cairo (AUC), New Cairo 11835, Egypt*

*^3^Associate to the UNESCO UNISA Africa Chair in Nanosciences & nanotechnology*

***** *Correspondence: Hamza Mohamed*

*hamza@aims.ac.za*

500000

400000

300000

200000

100000

0

-200

-100

0

**Apparent Zeta Potential (mV)**

100

200

**Total Counts**

**Fig. S1: Zeta potential spectrum of WO3 dispersed in deionized water measured at natural pH.**

14

12

10

8

6

4

2

0

0.1

1

10

100

1000

10000

**Size (d.nm)**

**Intensity (Percent)**

***Fig. S2: Particle size distribution spectrum of WO3 dispersed in deionized water measured at natural pH.***


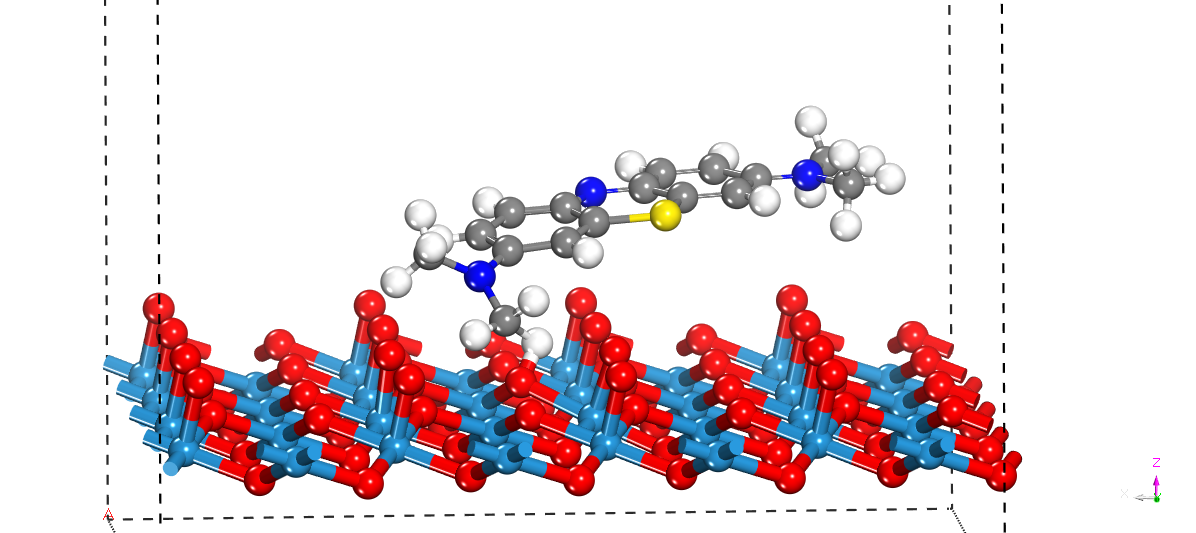


**Figure S3:** Adsorption of HC1 over Oxygen type 1


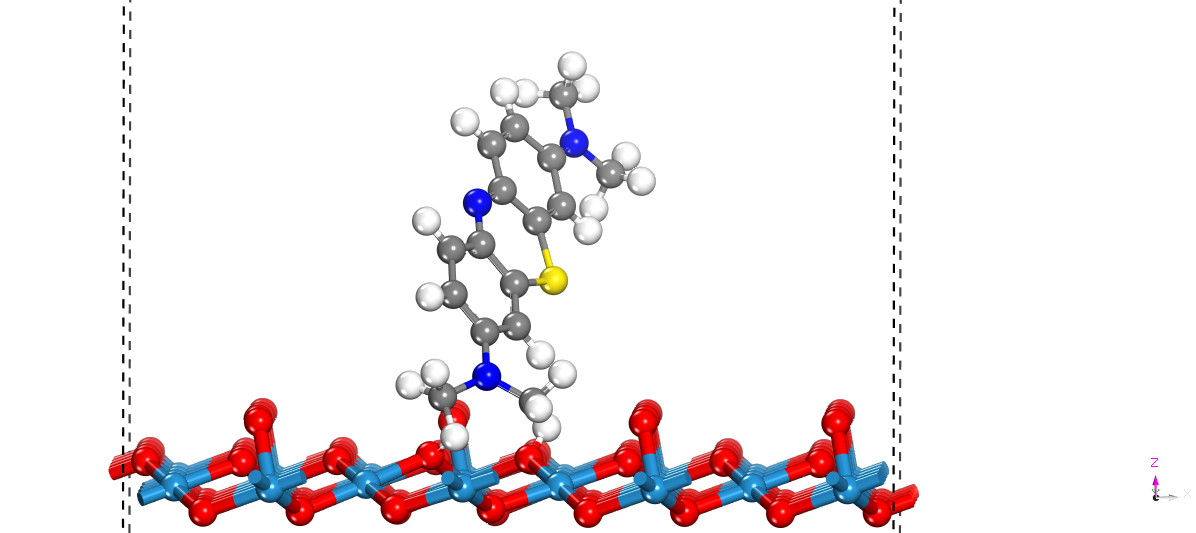


**Figure S4:** Adsorption of HC1 and HC2 over two atoms of Oxygen type 1


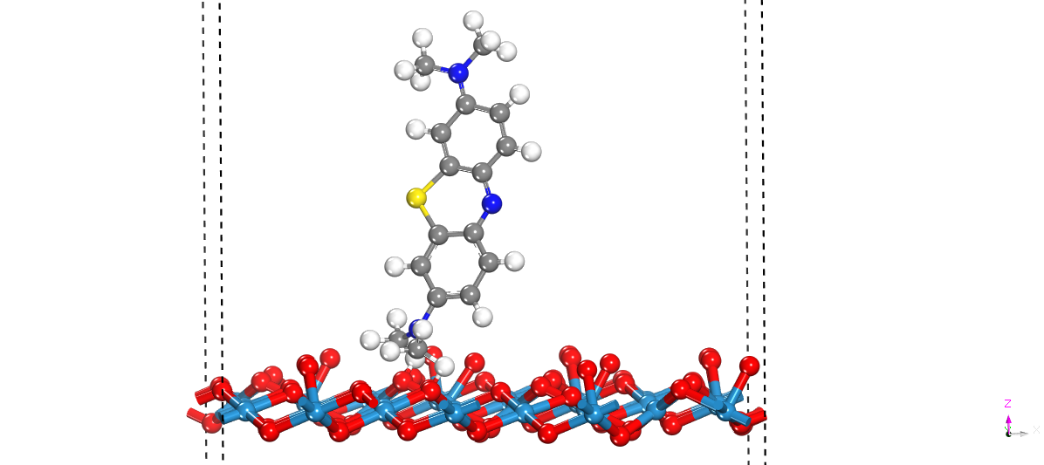


***Figure S5:*** *Adsorption of HC1 and HC2 over two atoms one of Oxygen type 1 and one of Oxygen type 2*


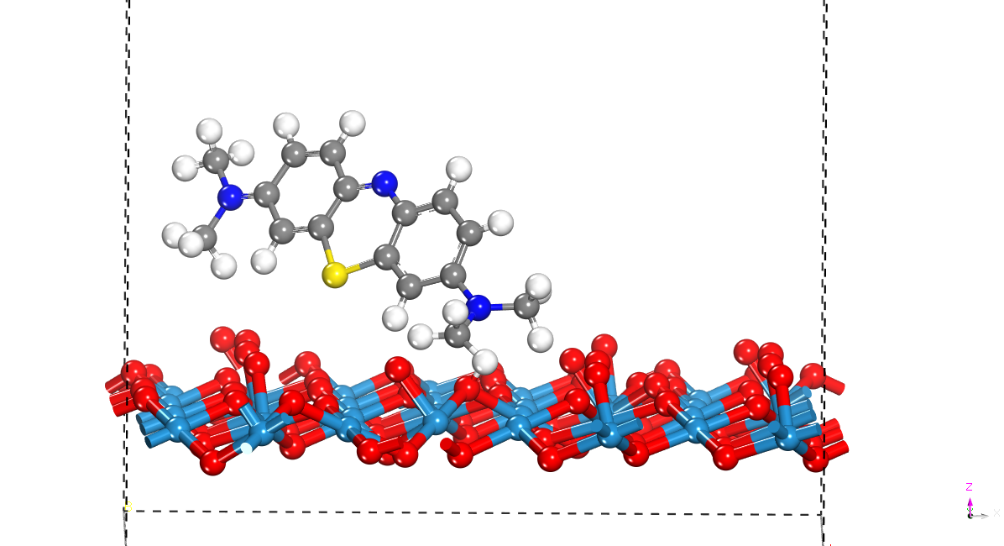


**Figure S6:** Adsorption of HC1 over one atom of Oxygen type 2


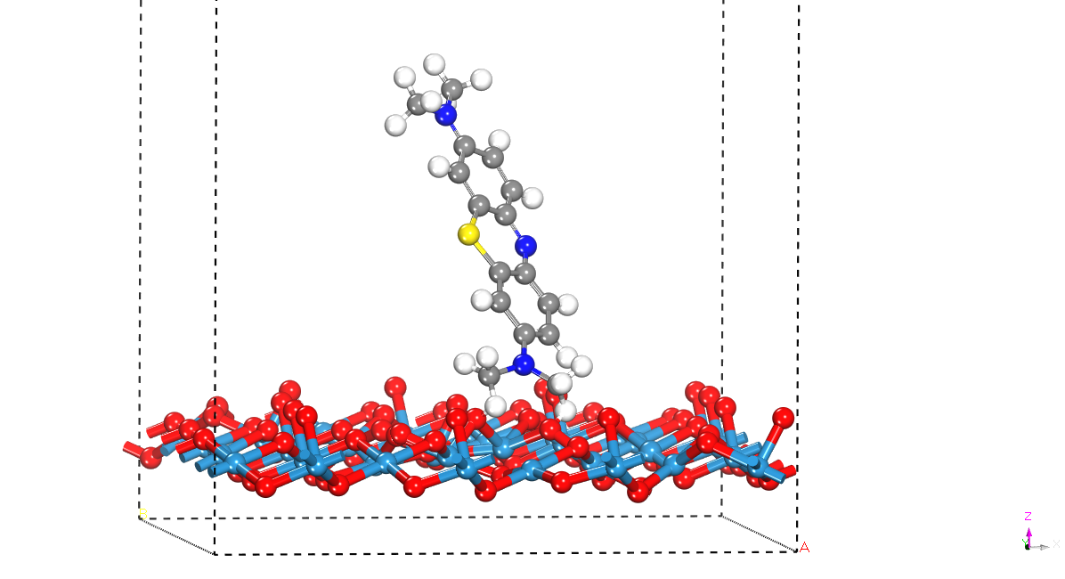


**Figure S7:** Adsorption of HC1 and HC2 over two atoms of Oxygen type 2


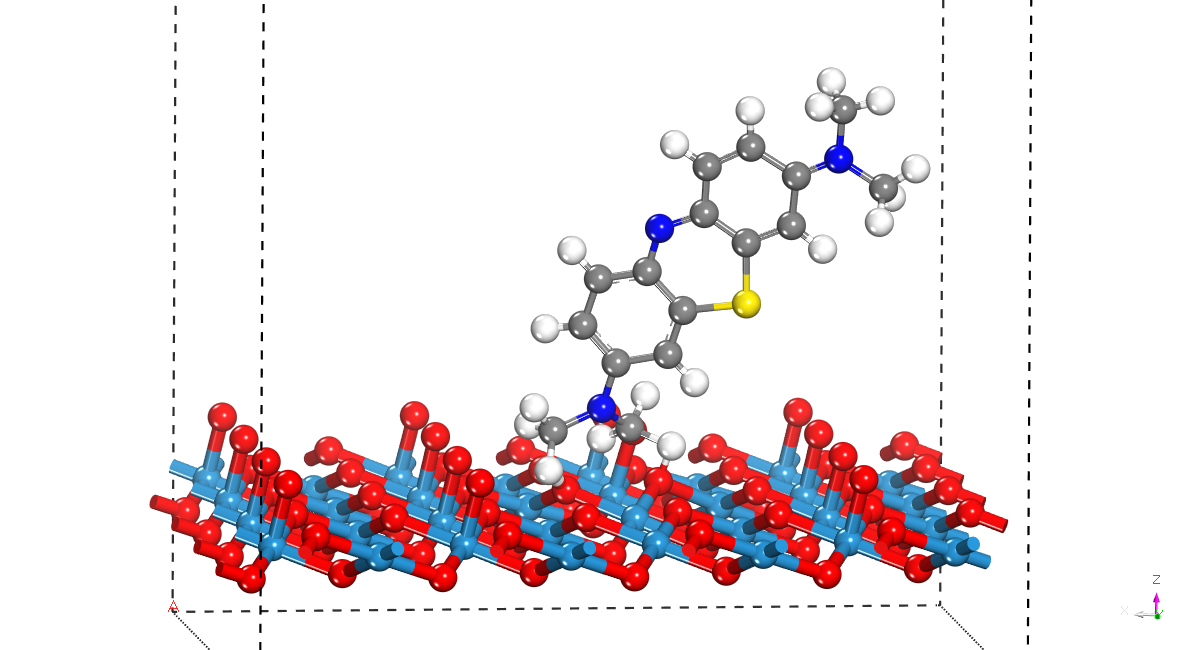


**Figure S8:** Adsorption of HC1 and HC2 over one atom of Oxygen type 1


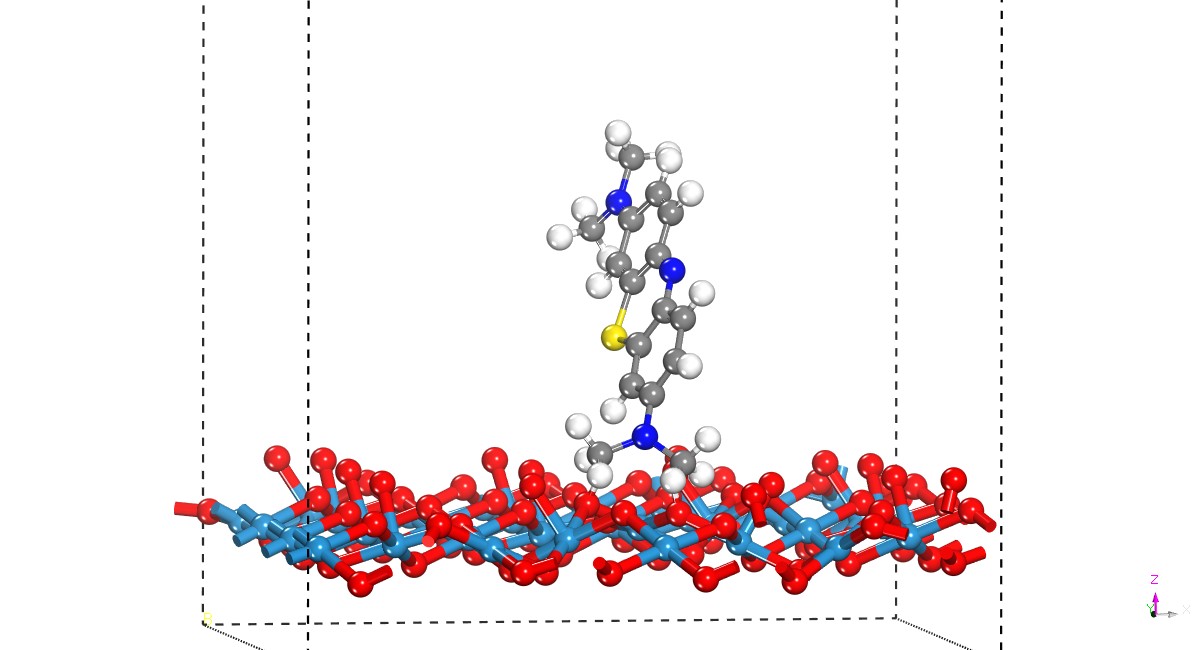


**Figure S9:** Adsorption of HC1 and HC2 over two atoms of Oxygen type 2


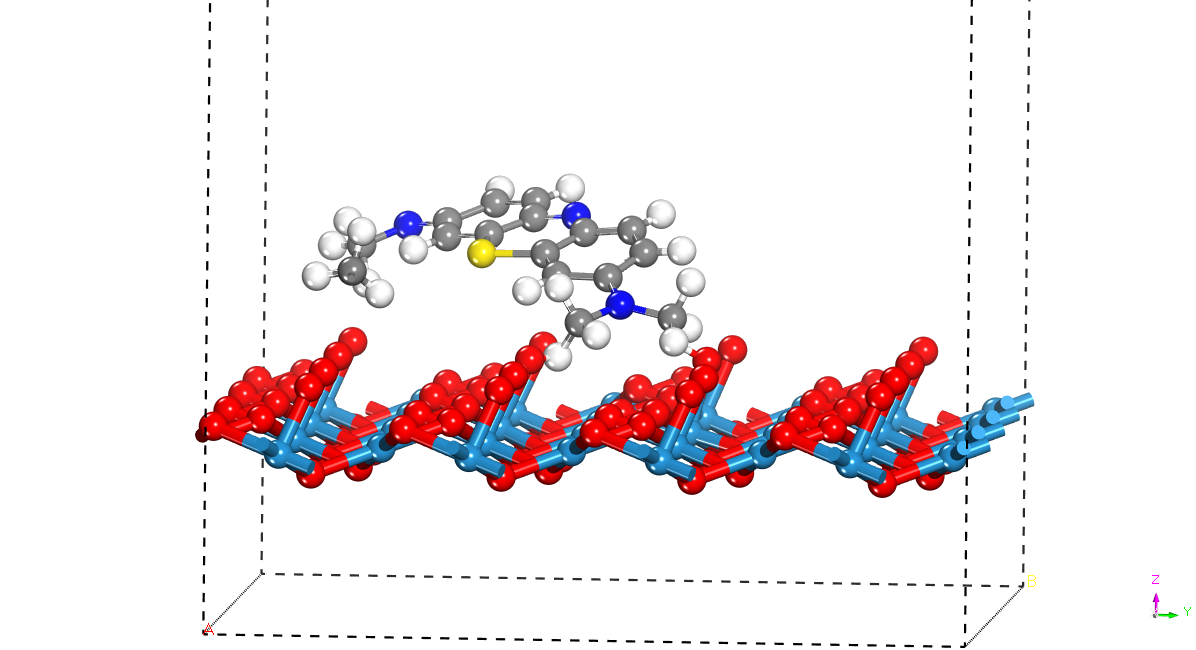


**Figure S10:** Adsorption of HC1 and HC2 over two atoms of Oxygen type 3


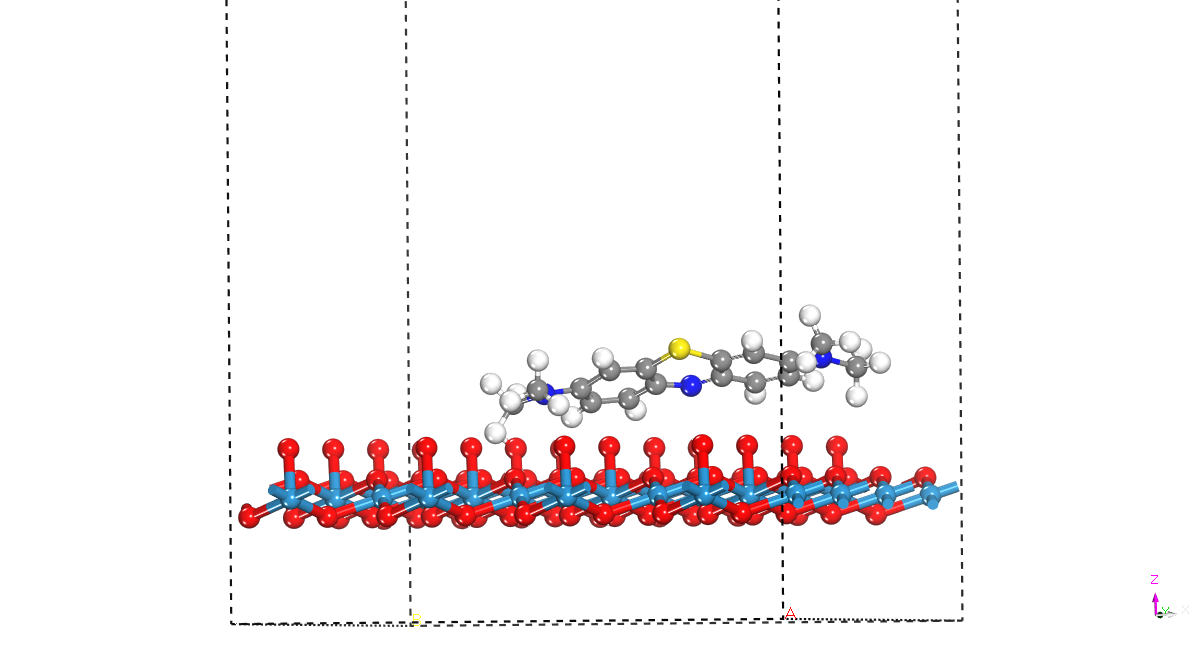


**Figure S11:** Adsorption of HC1 over one atom of Oxygen type 3


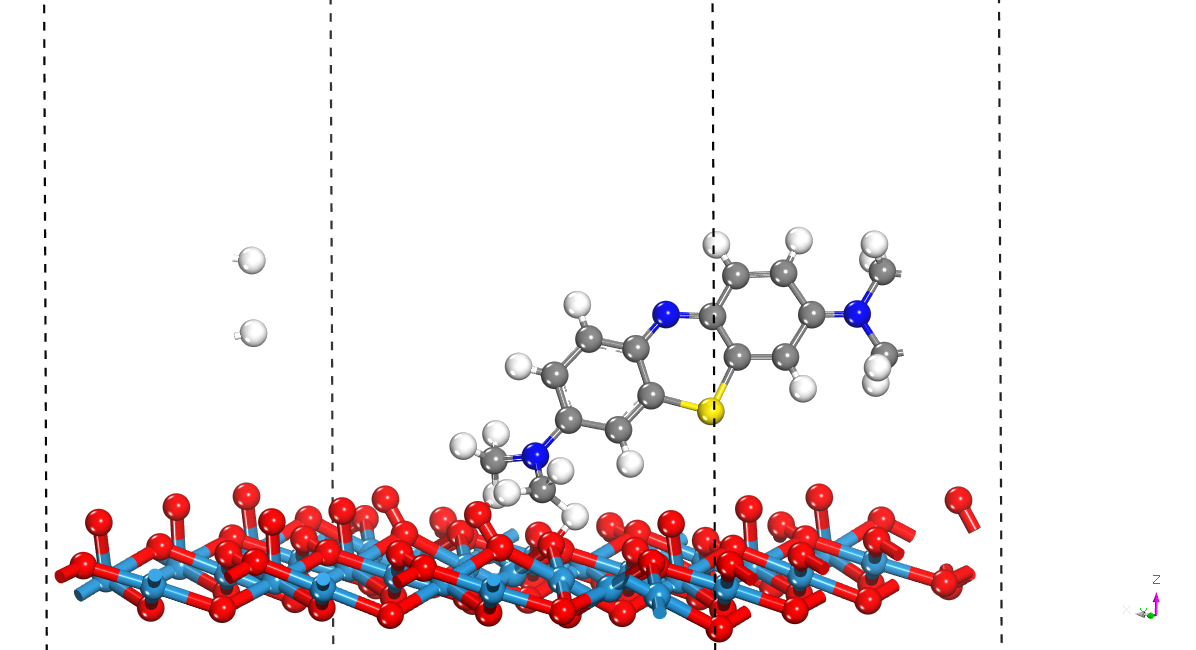


**Figure S12:** Adsorption of HC1 over two atoms of Oxygen type 1 and Type 3


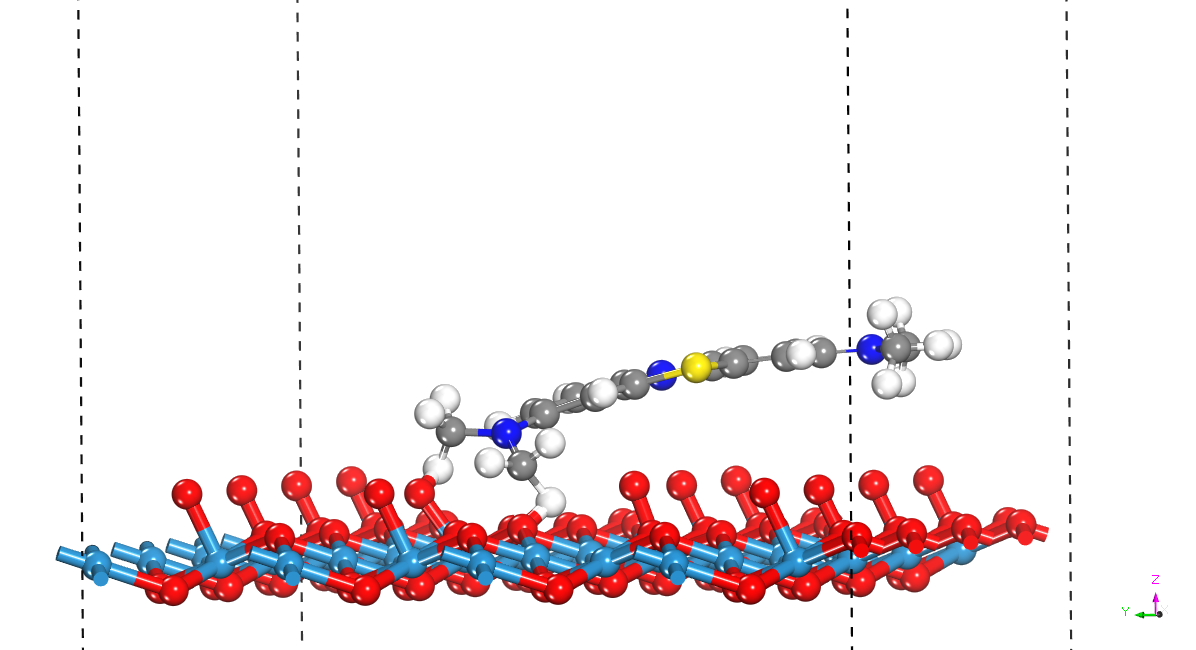


**Figure S13:** Adsorption of HC1 over two atoms of Oxygen type 2 and type 3


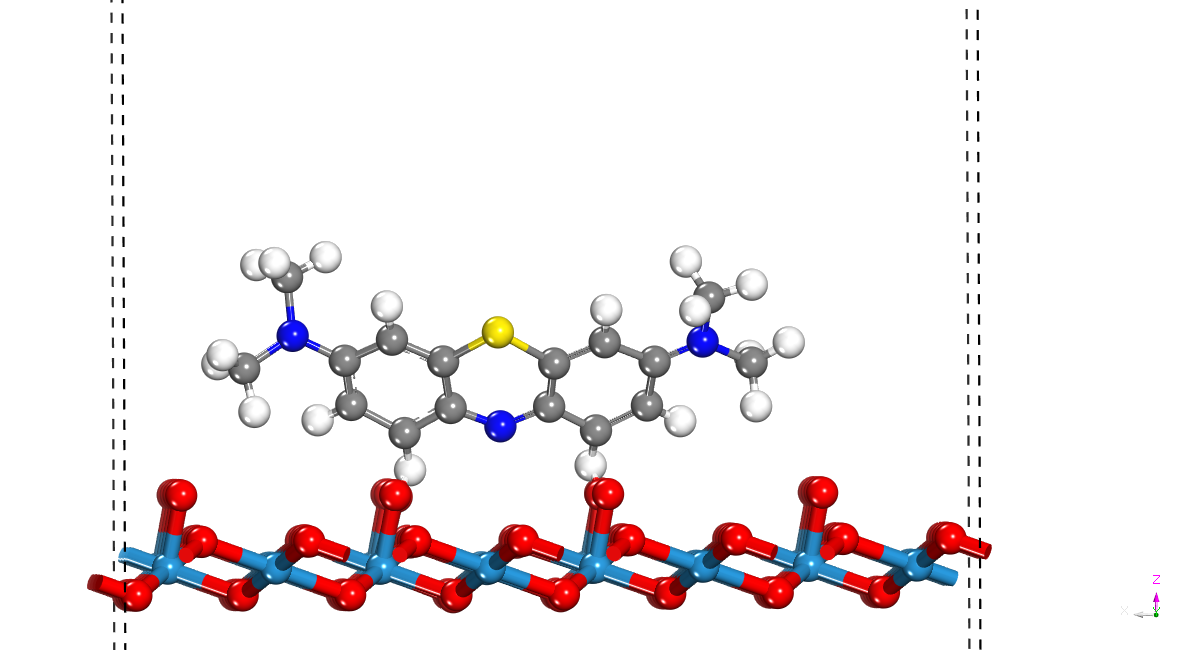


**Figure S14:** Adsorption of HC1 and HC3 over two atoms of Oxygen type 3
